# TriMaster: randomised double-blind crossover study of a DPP4 inhibitor, SGLT2 inhibitor and thiazolidinedione as second-line or third-line therapy in patients with type 2 diabetes who have suboptimal glycaemic control on metformin treatment with or without a sulfonylurea—a MASTERMIND study protocol

**DOI:** 10.1136/bmjopen-2020-042784

**Published:** 2020-12-21

**Authors:** Catherine Angwin, Caroline Jenkinson, Angus Jones, Christopher Jennison, William Henley, Andrew Farmer, Naveed Sattar, Rury R Holman, Ewan Pearson, Beverley Shields, Andrew Hattersley, MASTERMIND consortium

**Affiliations:** 1 Institute of Biomedical and Clinical Science, University of Exeter Medical School, University of Exeter, Exeter, Devon, UK; 2 Department of Mathematical Sciences, University of Bath, Bath, Somerset, UK; 3 Health Statistics Group, University of Exeter Medical School, University of Exeter, Exeter, UK; 4 Nuffield Department of Primary Care Health Sciences, University of Oxford, Oxford, UK; 5 Institute of Cardiovascular and Medical Sciences, University of Glasgow, Glasgow, UK; 6 Radcliffe Department of Medicine, University of Oxford Medical Sciences Division, Oxford, UK; 7 University of Dundee, Dundee, Dundee, UK

**Keywords:** diabetes & endocrinology, clinical trials, therapeutics

## Abstract

**Introduction:**

Pharmaceutical treatment options for patients with type 2 diabetes mellitus (T2DM) have increased to include multiple classes of oral glucose-lowering agents but without accompanying guidance on which of these may most benefit individual patients. Clinicians lack information for treatment intensification after first-line metformin therapy. Stratifying patients by simple clinical characteristics may improve care by targeting treatment options to those in whom they are most effective. This academically designed and run three-way crossover trial aims to test a stratification approach using three standard oral glucose-lowering agents.

**Methods and analysis:**

TriMaster is a randomised, double-blind, crossover trial taking place at up to 25 clinical sites across England, Scotland and Wales. 520 patients with T2DM treated with either metformin alone, or metformin and a sulfonylurea who have glycated haemoglobin (HbA_1c_) >58 mmol/mol will be randomised to receive 16 weeks each of a dipeptidyl peptidase‐4 inhibitor, sodium-glucose co-transporter-2 inhibitor and thiazolidinedione in random order. Participants will be assessed at the end of each treatment period, providing clinical and biochemical data, and their experience of side effects. Participant preference will be assessed on completion of all three treatments. The primary endpoint is HbA_1c_ after 4 months of therapy (allowing a range of 12–18 weeks for analysis). Secondary endpoints include participant-reported preference between the three treatments, tolerability and prevalence of side effects.

**Ethical approval:**

This study was approved by National Health Service Health Research Authority Research Ethics Committee South Central—Oxford A, study 16/SC/0147. Written informed consent will be obtained from all participants. Results will be submitted to a peer-reviewed journal and presented at relevant scientific meetings. A lay summary of results will be made available to all participants.

**Trial registration numbers:**

12039221; 2015-002790-38 and NCT02653209.

**Strengths and limitations of this study**

- This is the first blinded three-way crossover trial of glucose-lowering therapies in type 2 diabetes, allowing comparison of short-term treatment response and side effects across three agents within the same individuals.
- This study design enables assessment of stratification allowing for within-person variation in response.
- This will be the first study to assess patient preference for choosing between three glucose-lowering therapies.
- A limitation is that only short-term glycaemic response and side effects can be assessed in a study of this design.

## Background and rationale

In recent years the choice of therapies designed to lower glucose in patients with type 2 diabetes (T2DM) has increased^1^ but there remains limited information as to which patients may respond well, moderately or poorly to any of the treatment options.^2 3^ Treatment intensification is recommended in a stepwise approach, with guidelines usually including a number of different agents after metformin in those without established cardiac or renal disease.^2 4^

T2DM is a heterogenous condition and response to glucose-lowering therapy appears to vary substantially between individuals. Therefore, identification of subgroups of patients who respond well or poorly to a specific therapy, or with an altered risk of treatment-specific side effects, could improve targetting of treatment. This stratified approach to therapy is most likely to be successful if based on clinical characteristics and biomarkers that are readily available in routine clinical care: T2DM is common, most therapies are relatively inexpensive and most management is undertaken in primary care, therefore stratification based on expensive biomakers, or those with limited availability, is unlikely to be widely adopted.

A number of previous studies have shown simple clinical characteristics and biomarkers are associated with variation in glycaemic response for individual therapies.^5–8^ However, to be most useful for stratification a marker needs to predict differential response between therapies.^9^ Work by the MASTERMIND consortium using routine and trial data has strengthened the evidence that clinical features are associated with differential glycaemic response to dipeptidyl peptidase‐4 (DPP4)-inhibitors, sodium-glucose co-transporter-2 (SGLT2)-inhibitors and thiazolidinediones.^10–12^ Analysis of data from the UK Clinical Practice Research Datalink (CPRD) and a Diabetes Outcome Progression Trial (ADOPT) trial showed that sex and body mass index (BMI) above and below 30 were associated with differential glycaemia response between sulfonylureas and thiazolidinediones.^10^ In addition, individuals within a normal estimated glomerular filtration rate (eGFR) range, with a higher eGFR show a better glycaemia response to SGLT2 inhibitors while individuals with a lower eGFR may have a better glycaemic response when taking a DPP4 inhibitor (Janssen, personal communication from MASTERMIND industry group, 2014). The features identified (sex, obesity and renal function), are routinely measured at low cost, meaning potential stratification using these characteristics could be easily implemented in clinical practice.

TriMaster aims to test potential glycaemic therapy stratification in T2DM using response to three standard glucose-lowering agents. It will determine whether subgroups defined by routinely measured features respond to a greater or lesser degree (with regard to glycaemic change) to DPP4 inhibitors, SGLT2 inhibitors and thiazolidinediones, and provide a resource for further investigation of stratification between these therapies in the future.

These therapies were selected on the basis of differential response seen in pilot studies, and the choice of available oral third-line therapies at the start of the study.

### Hypotheses

The trial is designed with the following hypotheses:

1. Patients with insulin resistance, characterised clinically by a raised BMI (>30 kg/m^2^), compared with non-obese patients will: (i) respond well to pioglitazone, a thiazolidinedione that works as an insulin sensitiser^13^; (ii) respond less well to sitagliptin, a DPP4 inhibitor which works through stimulating endogenous insulin secretion post-prandially.^14^
2. Patients with modestly reduced eGFR (60–90 mL/min/1.73 m^2^), compared with those with eGFR >90 mL/min/1.73 m^2^ will: (i) respond less well to canagliflozin, an SGLT2 inhibitor, which works through inhibiting the active reabsorption of glucose in the proximal tube,^15^ as the reduced eGFR will reduce the glucose-lowering efficacy; (ii) respond well to sitagliptin, a DPP4 inhibitor that is renally cleared, as the reduced eGFR will increase plasma DPP4 inhibitor concentrations.

## Primary objectives

To test two hypotheses of drug response stratification based on drug mechanism of action and pharmacokinetics to answer the following clinical questions:

1. Do obese patients (BMI>30 kg/m^2^), compared with non-obese patients, achieve a lower glycated haemoglobin (HbA_1c_) when assigned to pioglitazone rather than sitagliptin?
2. Do patients with an eGFR 60–90 mL/min/1.73 m^2^ achieve a lower HbA_1c_, compared with patients with an eGFR >90 mL/min/1.73 m^2^, when assigned to sitagliptin rather than canagliflozin?

## Secondary objectives

The design of the study provides people with T2DM the unusual opportunity to try a panel of three available glucose-lowering therapies and to express a preference based on their experience of each. The study’s secondary objectives are to determine:

1. Patient treatment preference within hypothesised strata and overall.
2. Prevalence of side effects within hypothesised strata and for specific drugs, to include: weight gain, hypoglycaemia, oedema, genital tract infection and discontinuation of therapy.
3. Predefined test of sex heterogeneity with pilot data suggesting women are more likely to show an improved response relative to men for pioglitazone.
4. Tolerability of treatments within hypothesised strata and overall.

## Methods and analysis

We have used the Standard Protocol Items: Recommendations for Interventional Trials reporting guidelines in the design of the protocol and preparation of this paper^16^ (online supplemental appendix 1).

10.1136/bmjopen-2020-042784.supp1 Supplementary data

### Overview of trial design

**Figure 1:**
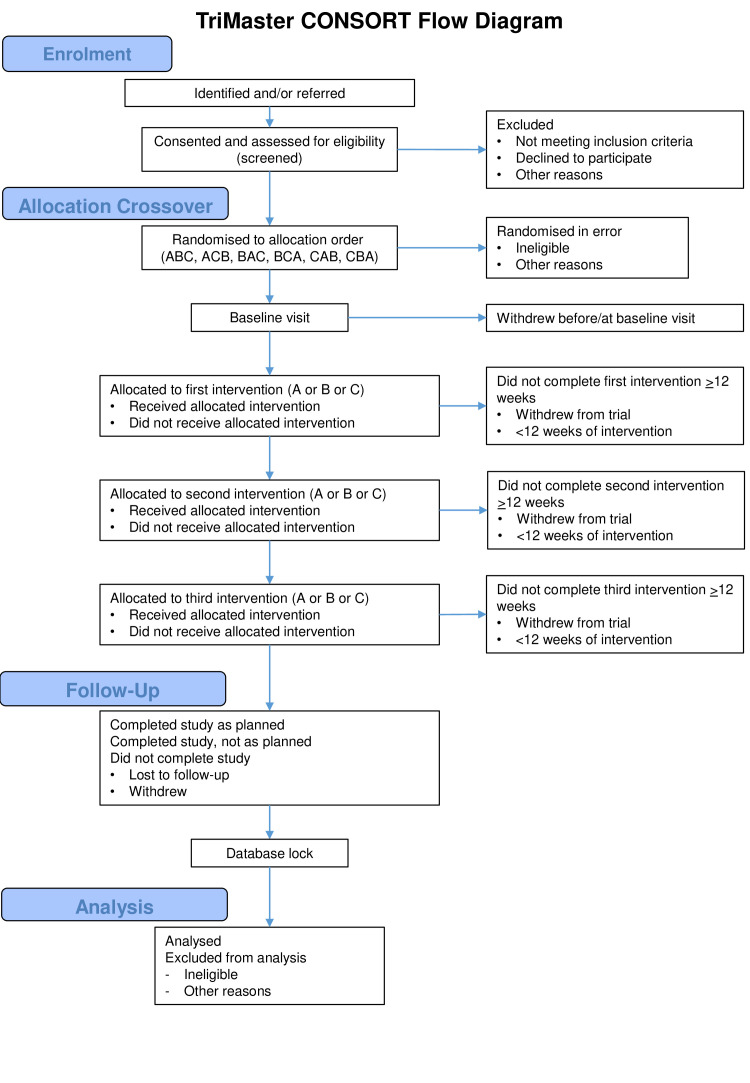
TriMaster consort diagram.

TriMaster is a phase IV, academically designed and run, multicentre, randomised, double-blind, 12-month crossover trial of a DPP4 inhibitor (sitagliptin), thiazolidinedione (pioglitazone) and SGLT2 inhibitor (canagliflozin) as a second-line or third-line therapy in patients with T2DM who have suboptimal glycaemic control on metformin alone or metformin and sulfonylurea (figure 1). The three-way crossover will be undertaken as an efficient, faster and more cost-effective approach to address both hypotheses, requiring fewer participants than performing two 2-way cross over studies.

Five hundred and twenty participants with T2DM will be recruited, aged 30–80 years on stable doses of metformin alone or metformin and a sulfonylurea with HbA_1c_>58 mmol/mol (>7.5%) and ≤110 mmol/mol (≤12.2%). Each participant will attend one screening and, if eligible, five research visits over a 12-month period (50–60 weeks max/min visit windows). They will receive the three blinded second-line and third-line oral therapies in random order for 16–18 weeks each, with no washout period between therapies. Participant feedback from pilot studies found repeated washout periods increased rates of withdrawal due to poorly tolerated hyperglycaemia. Once stopped, none of the three glucose-lowering agents used in this study have a continuing glucose-lowering effect beyond 4 weeks (all three drugs have half-lives between 7 and 14 hours so their effects should be negligible after a week)^17–19^ and HbA_1c_ measurement reflects glycaemia over the preceding 8 weeks to 12 weeks period. HbA_1c_ measurements taken after 16 weeks will therefore allow a 4-week ‘wash-in’ period and effectively reflect response to each treatment period.

Participants will each act as their own control, and on completion of all three treatments will be asked to rank the treatments taken in order of preference. Eligible participants will be recruited at 20–25 UK sites; the trial is sponsored by the Royal Devon and Exeter National Health Service (NHS) Foundation Trust and hosted at the National Institute for Health Research Exeter Clinical Research Facility. A full list of recruiting sites will be available via the ISRCTN registration.

### Eligibility criteria

All potential participants will undergo a formal screening visit to assess and confirm eligibility as listed in table 1.

**Table 1:** Eligibility criteria

Inclusion criteria	Exclusion criteria
Clinical diagnosis of T2DM. Age ≥30 and ≤80 years. Currently treated with one or two classes of oral glucose-lowering therapy (given either as separate or combined medications), that do not include a DPP4 inhibitor, a SGLT2 inhibitor or a thiazolidinedione. Diabetes duration ≥12 months. No change in diabetes treatment (new treatments or dose change) within previous 3 months. HbA_1c_ > 58 mmol/mol (>7.5%) and ≤110 mmol/mol (≤12.2%). eGFR ≥60 mL/min/1.73 m². Able and willing to give informed consent.	Changes in glucose-lowering therapy or dose within last 3 months. ALT >2.5×upper limit of the assay normal range or known liver disease, specifically bilirubin >30 μmol/L that is associated with other evidence of liver failure. Insulin treated within the last 12 months. Treated with study drugs within the last 3 months. Limb ischaemia shown by absence of both pulses in one or both feet. Currently treated with corticosteroids. Currently treated with rifampicin, gemfibrozil, phenytoin and carbamazepine. Active infection (any infection requiring antibiotics). Foot ulcer requiring antibiotics within previous 3 months. Recent (within 3 months) significant surgery or planned surgery (excluding minor procedures). Acute cardiovascular episode (angina, myocardial infarction, stroke, transient ischaemic episode) occurring within the previous 3 months. History of heart failure. Current use of loop diuretic therapy (furosemide or bumetanide). History of bladder carcinoma. Current/ongoing investigation for macroscopic haematuria. History of diabetic ketoacidosis. History of pancreatitis. Pregnant, breastfeeding or planning a pregnancy over the study period. Concurrent participation on another Clinical Trial of an Investigational Medicinal Product (CTIMP) where the IMP is currently being taken, or without sufficient washout period (five times the longest half-life of the study IMPs) and without consultation with the CTIMP research team. Unable or unwilling to give informed consent. Females of childbearing potential must be willing to use an effective method of contraception from the time consent is signed until 7 days after treatment discontinuation. A negative pregnancy test is required within 7 days prior to treatment initiation and will be required for continuation at each study visit.

ALT, alanine aminotransferase; DPP4, dipeptidyl peptidase‐4; eGFR, estimated glomerular filtration rate; HbA_1c_, glycated haemoglobin; SGLT2, sodium-glucose co-transporter-2; T2DM, type 2 diabetes mellitus.

### Outcome measures

In line with WHO guidelines, response to therapy will be assessed by measurement of HbA_1c_.^20^ The primary outcome is the HbA_1c_ value achieved after each 16-week treatment period. Should a participant be unable to complete a full 16-week treatment period, HbA_1c_ will be measured and included in the main analysis if the participant has taken the study drug for at least 12 weeks.

Secondary outcomes will be participant-reported preference between the three treatments, tolerability of the three treatments and prevalence of side effects. In addition, we will explore sex differences in response to the three drugs.

Participant willingness to continue a drug long-term will be recorded at the end of each treatment arm. Treatment preference, taken as a ranking of the three study drugs will be recorded at study completion. To inform this decision, in addition to their experience on each drug, clinical information including HbA_1c_ measurements and weight change will be fed-back to each participant. Frequency and severity of side effects will be recorded throughout the study alongside the Diabetes Treatment Satisfaction Questionnaire (DTSQ) to allow a formal validated assessment of participant satisfaction.

HbA_1c_ assessment during the study will be performed in local NHS laboratories to ensure results are available for screening, and to inform final patient preference. Central laboratory analysis will be undertaken at the Exeter Clinical Laboratory at the Royal Devon and Exeter NHS Foundation Trust for all other non-safety sample analysis. All analyses are routine biochemistry tests available in the NHS test repertoire. All assays are CE marked, fully validated and accredited by the UK Accreditation Service.

### Sample size

#### Primary outcome is HbA_1c_ at the end of each treatment period

This trial aims to test whether participants in a particular strata (S) respond differently to drug A and drug B compared with patients not in the strata (N). The primary outcome is the HbA_1c_ measurement after 4 months of each drug (table 2).

**Table 2:** Differences in response between two drugs and two strata in the crossover trial

Patient group	Drug A	Drug B	Difference
In strata (S)	HbA1c_SA_	HbA1c_SB_	HbA1c_SA_−HbA1c_SB_
Not in strata (N)	HbA1c_NA_	HbA1c_NB_	HbA1c_NA_−HbA1c_NB_

The null hypothesis is that the difference in achieved HbA_1c_ for the two drugs will be similar for the two groups of participants (ie, HbA1c_SA_−HbA1c_SB_=HbA1c_NA_−HbA1c_NB_).

HbA_1c_, glycated haemoglobin.

The null hypothesis is that the difference in achieved HbA_1c_ for the two drugs will be similar for the two groups of participants (ie, HbA1c_SA_−HbA1c_SB_=HbA1c_NA_−HbA1c_NB_ in table 2).

In a crossover trial of metformin vs repaglinide the SD of change in HbA_1c_ on the two different therapies was 8.7 mmol/mol.^21^ Analysis of CPRD showed obese patients respond better to thiazolidinediones (TZDs) and non-obese patients respond better to DPP4is, with an overall difference in response between strata of 3.1 mmol/mol (equivalent to 0.36SDs). Similarly, higher eGFR >90 mL/min/1.73 m² is associated with a better HbA_1c_ response to SGLT2i, while patients with an eGFR 60–90 mL/min/1.73 m² had a lower response to the SGLT2i and higher response to DPP4i with an overall difference in response between strata of 3.0 mmol/mol (equivalent to 0.35SDs) (Janssen, personal communication, 2014).

Using 90% power, alpha=0.05, to detect a difference of 0.35SDs we require 172 participants in each stratum, 344 in total. To allow for the possibility of unequal numbers in each stratum, the sample size has been increased to 358, assuming a 60:40 split (T2DM population CPRD 52:48 for both strata); a conservative withdrawal rate of 15% increases the study sample size to 422. To allow for participants excluded from primary analysis due to fewer than 12 weeks on one or more study drugs (estimated at 19%), we will increase the total sample size for the study to 520.

### Investigational medicinal product

Trial interventions were chosen in line with UK NICE (National Institute for Health and Care Excellence) guidelines for first and second intensification of drug treatment in patients with T2DM.^2^ The three drugs will be provided to participants in a blinded format and randomised order at the starting dose indicated in the British National Formulary; sitagliptin 100 mg, canagliflozin 100 mg, pioglitazone 30 mg.

Investigational medicinal products (IMPs) will be supplied directly to recruiting site pharmacies by Tayside Pharmaceuticals, Dundee, UK. Tablets will be over-encapsulated in a hard gelatin capsule so that the IMPs are near identical in size and colour, and packed into bottles and distributed to each recruitment site (see the Randomisation, allocation and blinding section for further details).

Participants will be instructed to take one capsule, once daily, alongside their existing diabetes treatment and usual medications. They will be given a Drug Information Sheet in place of a standard summary of product characteristics stating the expected side effects of all three treatments. To allow feasible visit windows and prevent participants running out of IMP, each IMP bottle will contain 126 capsules, the equivalent of 18 weeks’ medication.

Where a participant is unable to tolerate a therapy, they will move to the next IMP in their randomised order, providing they remain clinically safe to continue in the study. Dose modification, reduction or delay will not be permitted due to the blinded nature of the trial. Participants will be asked to return the IMP bottle and all unused capsules; research staff will perform a capsule count for adherence and accountability purposes.

### Randomisation, allocation and blinding

The study has six treatment sequence permutations: ABC, ACB, BAC, BCA, CAB and CBA; participants will be randomly allocated to one of the six sequences when confirmed as eligible in the study database. The study is double-blind and all clinical, participant and laboratory assessments will be made prior to database lock, final analysis and unblinding of the drug order.

A block randomisation list (block size 12) will be created using Statsdirect by the Trial Statistician and the randomisation seed recorded. The randomisation list will be provided to the study database team who will randomly allocate blocks of 12 to each of the recruitment sites (to ensure balance between the six treatment orders at each site), with the allocation remaining blinded to the rest of the study team. The IMP supplier will provide each site with blocks of 12 drugs (four of each of the three study drugs) with the 12 bottle IDs labelled in random order to avoid the drug type being easily identifiable. The bottle IDs and contents assigned to each recruitment site will be recorded in the study database and accessible only to the database team.

To ensure allocation concealment, randomisation will be centralised via the study database. Eligibility will be confirmed by research teams and the participant randomised to a blinded treatment order, allocated by the study database. Prior to research visits one, two and three, the database will allocate the next available IMP bottle of the correct drug type held at that site, according to this treatment order. Study prescriptions detailing the allocated bottle ID will be processed and dispensed by the site clinical trials pharmacy.

Participants will not be recruited against specific strata; BMI and eGFR defined stratum will be monitored as recruitment and randomisation progresses. Data on the distribution will be provided to the Data Monitoring Committee (DMC) and if enrolment is unevenly distributed to an extent that the study hypotheses cannot be robustly tested, the relevant stratum may be ‘switched off’ by the data programmer to prevent further randomisation into the relevant strata.

Participants who withdraw before randomisation will be replaced. However, once randomised, their data will be included in analysis. To maintain data quality and trial integrity, unblinding via code breaks will occur only in exceptional circumstances where knowledge of the IMP is deemed essential for the correct clinical management of the participant, a medical emergency where someone other than the participant has taken the IMP, or where this information is needed to establish expectedness of a potential Suspected Unexpected Serious Adverse Reaction. The emergency code break table is available electronically on the study database requiring multiple confirmation steps to avoid accidental unblinding, and on paper in the central coordinating centre. In the event a code break is required this will be done by a member of staff independent of the main trial team. A study involvement card with study ID, IMP details, and contact information for local and central emergency unblinding will be provided to all participants.

### Study visits and procedures

**Figure 2:**
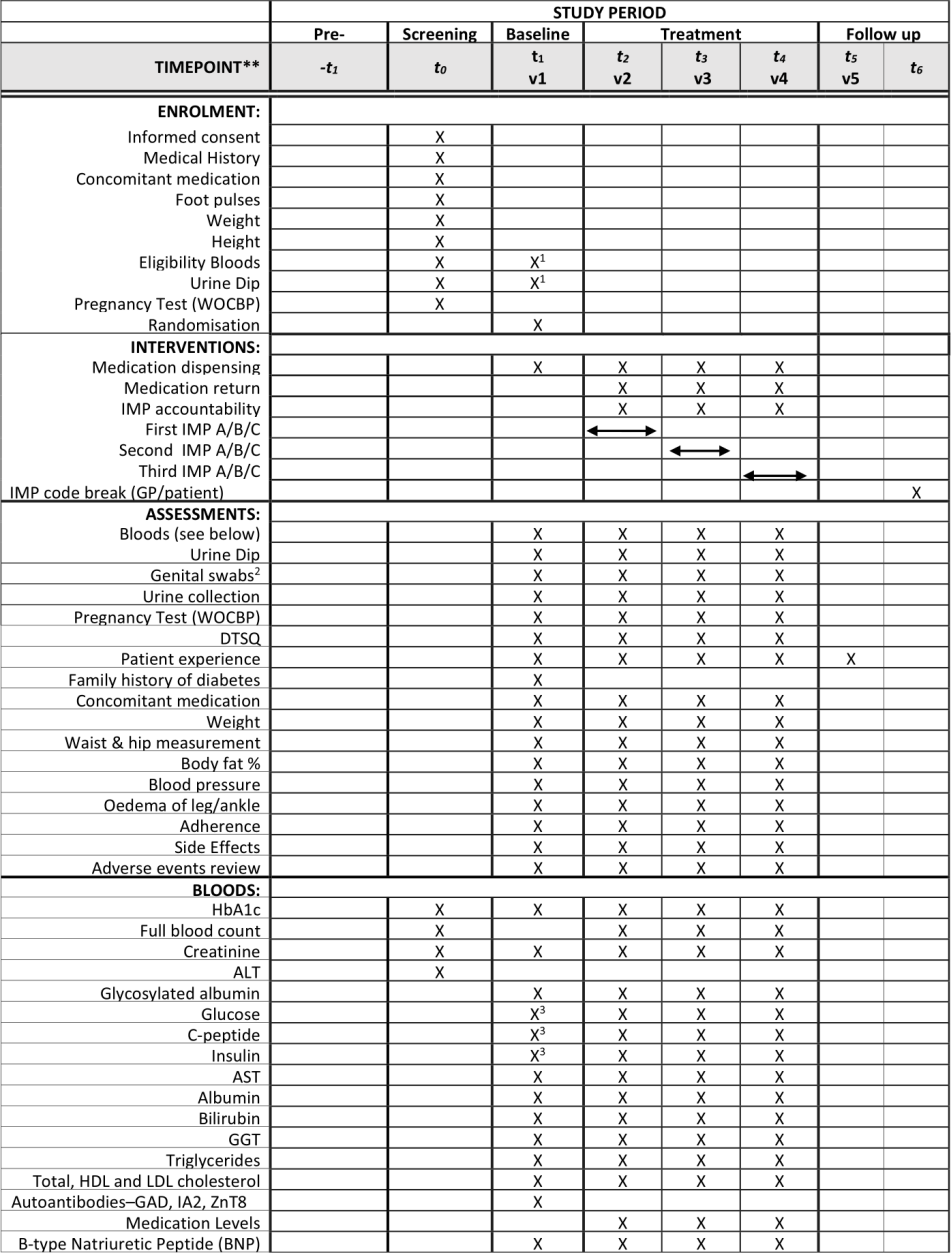
TriMaster schedule of assessment. ^1^Where baseline visit takes place more than 2 weeks after screening visit, eligibility blood samples must be repeated. ^2^Optional procedure for participants at sites which have previously agreed to sample collection. ^3^Analysis performed on both visit 1 baseline and visit 1 mixed-meal tolerance test samples. Other analyses at visit 1 performed on baseline only. DTSQ, Diabetes Treatment Satisfaction Questionnaire; HbA_1c_, glycated haemoglobin; IMP, investigational medicinal product

Figure 2 illustrates the schedule of assessments.

### Identification and recruitment

Potential participants will be identified through primary and secondary care, research databases and direct clinician referral, provided with an information sheet, and invited to attend a screening visit. Following informed consent (online supplemental appendix 2) by trained and delegated research staff, clinical characteristics (height, weight, waist and hip circumference), medical history, concomitant medication details and non-fasting blood samples will be collected to confirm eligibility. Eligible participants will be randomised into the trial, assigned a unique study ID and allocated a drug order.

10.1136/bmjopen-2020-042784.supp2 Supplementary data

### Baseline visit

Within 2 weeks of screening, participants will attend a fasting baseline research visit. Baseline physiological data will be collected, along with self-reported compliance to existing diabetes medication. Participant’s personal priorities in choosing between treatment options and experience of side effects on current treatment will also be recorded. Participants will have underlying pathophysiology assessed in a 2-hour mixed-meal tolerance test using a standard meal drink (Fortisip). The meal test will be undertaken using 250 mL of Nutricia Fortisip or 160 mL of Nutricia Fortisip Compact. Equivalent products may be used where Fortisip cannot be tolerated. Blood and urine samples will be collected for analysis and future biomarker discovery at baseline, and then at 30 min intervals (0, 30, 60, 90 and 120 min) following the meal drink. Participants at the central Exeter site will also be invited to provide a self-collected genital swab sample to identify development of subclinical colonisation of candida or bacteria.

Subsequent research visits will take place after 16–18 weeks of study treatment. However, patients will be offered the opportunity to stop a treatment early and move onto the next treatment period if they are unable to tolerate the therapy. Visits will repeat the baseline physiological measurements with samples collected at a single time point. Participants will provide fasting blood samples for immediate measurement of HbA_1c_, and subsequent assessment to include fasting glucose, c-peptide, insulin, glycosylated albumin, creatinine, lipid profile and drug levels. Weight, blood pressure, adherence and data about patient experience will also be collected, including perceived side effects, preparedness to remain on the drug long-term and health-related quality of life. Where collected at baseline, subsequent genital swabs will be repeated at study visits two to four.

Case report forms will be completed at recruiting centres and securely transferred to the central team via nhs.net email. OpenText TeleForm will be used for data capture and transfer to the study database. Identifiable data will be securely stored at recruiting centres, research data transferred to Exeter will be accessed only by delegated members of the research team.

### Questionnaires: participant preference

On completion of the third study drug, participants will be provided with a summary of their previous assessments of each therapy. At a final study visit, participants will first rank the study drugs based solely on their own experience on treatment. HbA_1c_ and weight data for each drug period will then be provided by the research team, and a repeat ranking recorded. This summary assessment was developed with the TriMaster Patient Involvement Group and the Peninsula Research Bank Lay Committee members. Endpoints of willingness to remain on study drug long-term, and impact on daily life were identified as the best representations to capture participant preference for the study.

A final version of drug preference and clinical data will be provided to the participant and their clinician. This document, provided directly by the Exeter CTU team to ensure research teams remain blinded throughout, will contain details of the unblinded study drugs A, B and C to inform future treatment choices. All study procedures will occur within the 50–60-week trial period but permission will be requested to contact participants after primary analysis is complete to assess future treatment choice.

Participants will also complete the DTSQ at baseline and the change version (DTSQc) after each treatment period to collect validated satisfaction scores.

### Statistical analysis

Analyses and reporting will follow Consolidated Standards of Reporting Trials guidance for randomised crossover trials.^22^ This study is not designed to test drug efficacy but the effectiveness of stratification. Therefore, only patients completing at least 12 weeks on therapy (sufficient to allow HbA_1c_ to reflect glycaemia control on the drug) will be included in primary analysis. In addition, we will perform a secondary analysis of tolerability examining whether the proportion of participants completing at least 12 weeks differs for each drug, both within strata and overall.

Prior to main analysis, we will determine whether there is any evidence of carryover or period effects. Any carryover effect identified will be reported but not adjusted for in subsequent analysis. Period effects will be reported and adjusted for. We do not anticipate treatment effect carryover and have designed the study to limit potential carryover (as far as possible). Any period effect in the maximum 8 months between on treatment HbA_1c_s is likely to be minimal as mean progression is 1.0 mmol/mol/year (E Pearson, personal communication, data from GoDARTs population data).

There will be two primary analyses, one for each of the study hypotheses. For each hypothesis the primary analysis will be to assess whether the difference in achieved HbA_1c_ measurements for the two drugs is similar for the two groups of participants. The two hypotheses will be tested separately using linear mixed effects models to compare the strata on the two drugs of interest, with a random effects term for the participant. The key contrast of interest is the drug*strata interaction, where the strata is either obesity group or eGFR group. To determine whether there is a difference between drug classes in terms of the overall achieved HbA_1c_ after 4 months on each of the drugs, we will fit an additional model. Drug will be a factor and coded as a dummy variable as the comparison will be across three rather than two drug classes. Least square means will be extracted for the three drugs. Similar analysis will be carried out with weight after 4 months as the outcome.

In addition, we will examine the distribution of side effects reported across each of the three drugs. However, given the total numbers reporting each individual side effect will likely be small, we anticipate this will largely be descriptive, examining proportion of side effects observed with each drug.

For analysis of patient preference, we will only analyse the dataset where the participants have tried all three drugs. The mean rank for each drug will be calculated and tested against the null hypothesis that there is not a preferred drug and therefore the expected value of the rank for a given drug will be two. Further investigation of patient preference will be exploratory.

Reasons for missing data will be documented and the baseline characteristics of those with and without missing data compared.

### Monitoring

Due to the nature of standard diabetes treatments, it is expected that participants will experience some mild adverse events or reactions. These will be recorded at research visits and reported on a study-wide basis to the sponsor and DMC at regular intervals.

Serious adverse reactions where the IMP is assessed as having possible, probable or definite causality will be unblinded to enable full evaluation of expectedness in the context of the relevant safety profile. Independent auditing of the trial will be arranged by the sponsor, in addition to sponsor review.

### Oversight committees

The study will be run by a Trial Management Group with oversight from an independent Trial Steering Committee and DMC. These committees comprise independent experts in diabetes and statistical methodology and patient representatives and will meet regularly to monitor the scientific integrity and safety of the trial and provide independent advice. To ensure the safety of participants, the DMC will review unblinded safety data.

## Ethics and dissemination

This study has been reviewed and received ethics approval from the NHS Health Research Authority (HRA) Research Ethics Committee South Central—Oxford A, study 16/SC/0147. The clinical trial application was reviewed and approved by the UK Medicines and Healthcare products Regulatory Agency (MHRA) under EudraCT reference. All substantial and non-substantial amendments have received approval from HRA, REC and MHRA before implementation. The protocol has been registered with ClinicalTrials.gov and ISRCTN (trial registration dataset in online supplemental appendix 3).

10.1136/bmjopen-2020-042784.supp3 Supplementary data

All participants will be provided verbal and written information about the study prior to providing written informed consent and will be free to withdrawn at any time without affecting current or future clinical treatment.

### Changes to protocol

The study was first registered with Clinicaltrials.gov on 12 January 2016 and ISRCTN on 02 November 2016. Approved protocol amendments are in table 3.

**Table 3:** Protocol amendments

Protocol amendments	
**SA1** v3 06.07.16	Amendment to randomisation process to allocate individual bottles rather than ‘packs’ of 3 bottles to allow for shorter expiry dates, and clarification of safety reporting procedures.
**SA4** v4 20.03.17	Amendment to exclusion criteria to allow patients who have previously tried the study drugs to be included, as long as this has not been in the previous 3 months. The original criteria were unnecessarily strict and did not reflect real-world prescribing habits. The amendment also removed the blanket exclusion for patients in concurrent clinical trials, providing sufficient washout period between IMPs.
**SA6** v5 01.08.17	Amendment to eligibility criteria to include patients taking metformin-only, or metformin and a sulfonylurea. This was adjusted due to the change in guidelines and prescribing trends leading to decline in use of sulfonylureas. At the time of study design sulfonylureas were the most commonly prescribed second line therapy in the UK. Subsequent decline in their use in favour of DPP4 inhibitors and SGLT2 inhibitors,^2^ resulted in the inclusion of patients currently treated with either metformin and sulfonylureas or metformin only. We will perform a sensitivity analysis to determine if the difference in study ‘epoch’ (before/after this amendment) has any impact on the main study outcomes. Altered exclusion criteria also added ‘limb ischaemia’ due to updated safety information for canagliflozin, and an upper limit of HbA_1c_>110 mmol/mol.
**SA9** v6 15.05.18	Amendment to sample size due to over-cautious sample calculations (alpha changed to 0.05), extension to recruitment period due to delays in regulatory approvals at study set-up and slow early recruitment, and additional secondary analysis included on the advice of the Data Monitoring Committee.
**SA10** v7 22.02.19	Amendment to study analysis plan. Following advice from the Trial Steering Committee statistician, the protocol was amended to analyse only those completing at least 12 weeks on therapy, as this will determine whether the strata result in differences in response (we cannot adequately measure glycaemic response by HbA_1c_ if the patient has been on the drug for less than 12 weeks). A separate analysis will be performed to determine whether the strata influence tolerability by assessing whether the proportion completing at least 12 weeks on therapy differs by drug and strata.
**SA12** v8 20.03.20	Amendment to ensure ongoing participant safety and study integrity during COVID-19 pandemic. Urgent safety measures included (i) extension of visit windows to 14–18 weeks to allow greater flexibility for participants who are unwell/isolating, (ii) provision for remote visits with sample collection outside the usual research setting, (iii) ensuring participants remained on study therapy when only a remote visit is possible, by allowing an additional ‘continuation’ bottle of the same IMP to be issued, or when no other option, transfer to the next IMP without collection of blood samples.

DPP4, dipeptidyl peptidase‐4; HbA_1c_, glycated haemoglobin; IMP, investigational medicinal product; SGLT2, sodium-glucose co-transporter-2.

### Dissemination

Data and results related to protocol-derived outcomes will be published in peer-reviewed journals by the chief investigator on behalf of the MASTERMIND consortium and presented at scientific meetings. Anonymous trial data will be shared within the MASTERMIND consortium and after publication of results, data will be securely deposited in Exeter’s institutional repository and made available on request via the consortium’s data access group. A lay summary will be provided to all study participants and made available on the study website, and public registries.

### Patient and public involvement

Patients were involved in the design and conduct of this study. Following pilot studies, the TriMaster Patient Involvement Group provided feasibility feedback on the study design and the outcome measures used to record patient preference and experience of the study drugs. This group and the Peninsula Research Bank Lay Committee members assisted in the design of patient-facing documents, including consent forms, study and drug information sheets and data collection forms to assess patient preference.

## Supplementary Material

Reviewer comments

Author's manuscript

## References

[1] Qaseem A, Barry MJ, Humphrey LL, et al Oral pharmacologic treatment of type 2 diabetes mellitus: a clinical practice guideline update from the American College of physicians. Ann Intern Med 2017;166:279–90. 10.7326/M16-1860 28055075

[2] Dennis JM, Henley WE, McGovern AP, et al Time trends in prescribing of type 2 diabetes drugs, glycaemic response and risk factors: a retrospective analysis of primary care data, 2010-2017. Diabetes Obes Metab 2019;21:1576–84. 10.1111/dom.13687 30828962 PMC6618851

[3] Pearson ER Personalized medicine in diabetes: the role of 'omics' and biomarkers. Diabet Med 2016;33:712–7. 10.1111/dme.13075 26802434 PMC4879510

[4] Davies MJ, D'Alessio DA, Fradkin J, et al Management of hyperglycaemia in type 2 diabetes, 2018. A consensus report by the American diabetes association (ADA) and the European association for the study of diabetes (EASD). Diabetologia 2018;61:2461–98. 10.1007/s00125-018-4729-5 30288571

[5] Jones AG, McDonald TJ, Shields BM, et al Markers of β-cell failure predict poor glycemic response to GLP-1 receptor agonist therapy in type 2 diabetes. Diabetes Care 2016;39:250–7. 10.2337/dc15-0258 26242184 PMC4894547

[6] DeFronzo RA, Stonehouse AH, Han J, et al Relationship of baseline HbA1c and efficacy of current glucose-lowering therapies: a meta-analysis of randomized clinical trials. Diabet Med 2010;27:309–17. 10.1111/j.1464-5491.2010.02941.x 20536494

[7] Thong KY, McDonald TJ, Hattersley AT, et al The association between postprandial urinary C-peptide creatinine ratio and the treatment response to liraglutide: a multi-centre observational study. Diabet Med 2014;31:403–11. 10.1111/dme.12367 24246138

[8] Bihan H, Ng WL, Magliano DJ, et al Predictors of efficacy of GLP-1 agonists and DPP-4 inhibitors: a systematic review. Diabetes Res Clin Pract 2016;121:27–34. 10.1016/j.diabres.2016.08.011 27622682

[9] Dennis JM Precision medicine in type 2 diabetes: using individualized prediction models to optimize selection of treatment. Diabetes. In Press 2020;69:2075–85. 10.2337/dbi20-0002 32843566 PMC7506836

[10] Dennis JM, Henley WE, Weedon MN, et al Sex and BMI alter the benefits and risks of sulfonylureas and thiazolidinediones in type 2 diabetes: a framework for evaluating stratification using routine clinical and individual trial data. Diabetes Care 2018;41:1844–53. 10.2337/dc18-0344 30072404 PMC6591127

[11] Dennis JM, Shields BM, Hill AV, et al Precision medicine in type 2 diabetes: clinical markers of insulin resistance are associated with altered short- and long-term glycemic response to DPP-4 inhibitor therapy. Diabetes Care 2018;41:705–12. 10.2337/dc17-1827 29386249 PMC6591121

[12] Rodgers L, ER. P, Hammersley S, et al Patients with a high fasting glucose respond better to sulphonylureas than dipeptidylpeptidase IV (DPP-IV) inhibitors: a mastermind study. Diabet Med 2015;32.

[13] Viberti G, Kahn SE, Greene DA, et al A diabetes outcome progression trial (adopt): an international multicenter study of the comparative efficacy of rosiglitazone, glyburide, and metformin in recently diagnosed type 2 diabetes. Diabetes Care 2002;25:1737–43. 10.2337/diacare.25.10.1737 12351470

[14] Drucker DJ, Nauck MA The incretin system: glucagon-like peptide-1 receptor agonists and dipeptidyl peptidase-4 inhibitors in type 2 diabetes. Lancet 2006;368:1696–705. 10.1016/S0140-6736(06)69705-5 17098089

[15] Tahrani AA, Barnett AH, Bailey CJ Sglt inhibitors in management of diabetes. Lancet Diabetes Endocrinol 2013;1:140–51. 10.1016/S2213-8587(13)70050-0 24622320

[16] Chan A-W, Tetzlaff JM, Altman DG, et al Spirit 2013 statement: defining standard protocol items for clinical trials. Ann Intern Med 2013;158:200–7. 10.7326/0003-4819-158-3-201302050-00583 23295957 PMC5114123

[17] Merck Sharp D Summary of product characteristics: Januvia (sitagliptin phosphate monohydrate); electronic medicines compendium (EMC), 2020 Available: https://www.medicines.org.uk/emc/product/7887/smpc

[18] Napp Pharmaceuticals L Summary of product Characterstics: Invokana (canagliflozin hemihydrate); electronic medicines compendium (EMC), 2020 Available: https://www.medicines.org.uk/emc/product/8855/smpc

[19] Takeda Summary of product characteristics: Actos tablets, electronic medicines compendium (EMC), 2019 Available: https://www.medicines.org.uk/emc/product/1287/smpc

[20] WHO Use of glycated haemoglobin (HbA1c) in the diagnosis of diabetes mellitus, 2011 Available: www.who.int/diabetes/publications/report-hba1c_2011.pdf 10.1016/j.diabres.2011.06.025 21820751

[21] Lund SS, Tarnow L, Stehouwer CDA, et al Targeting hyperglycaemia with either metformin or repaglinide in non-obese patients with type 2 diabetes: results from a randomized crossover trial. Diabetes Obes Metab 2007;9:394–407. 10.1111/j.1463-1326.2007.00713.x 17391168

[22] Dwan K, Li T, Altman DG, et al Consort 2010 statement: extension to randomised crossover trials. BMJ 2019;366:l4378. 10.1136/bmj.l4378 31366597 PMC6667942

