# Supplementary material for: TriMaster: randomised double-blind crossover study of a DPP4 inhibitor, SGLT2 inhibitor and thiazolidinedione as second-line or third-line therapy in patients with type 2 diabetes who have suboptimal glycaemic control on metformin treatment with or without a sulfonylurea—a MASTERMIND study protocol
Source: BMJ Open. 2020 Dec 21;10(12):e042784. doi: 10.1136/bmjopen-2020-042784 (PMC7754630; doi:10.1136/bmjopen-2020-042784)
Supplement: Supplementary data [file bmjopen-2020-042784supp002.pdf]

Sponsored by the Royal Devon and Exeter NHS Foundation Trust

University of Exeter | Medical School

Site Logo(s)

STUDY NAME:
TRIMASTER

Screening ID:	Study ID:	DOB / / 19

CONSENT STATEMENTS	Please circle	Initials
I have been given study information leaflet [Version [ ] dated [ ]] and supplementary information about the treatments used in this study. I have had the opportunity to ask questions and have had these answered satisfactorily.	YES/NO	
I am happy to:<br>• attend 6 appointments, having fasted overnight for 4 of these.<br>• try 3 different regularly prescribed diabetes treatments for up to 16 weeks each.<br>• provide information about my diabetes for use in this project.<br>• allow the research team to contact my clinicians/GP about my diabetes treatment and study participation, and to provide them with clinical results relevant to my care.<br>• provide blood and urine samples for use by this study.	YES/NO	
I understand that:<br>• my participation is voluntary and that I may withdraw at any time without my clinical care being affected.<br>• individuals from the study team, regulatory authorities or the NHS Trust will have access to relevant sections of my medical notes and data collected during the study for research, monitoring and audit purposes.	YES/NO	
**OPTIONAL CONSENT STATEMENTS**		
I agree that DNA may be extracted from samples for the purpose of this project	YES/NO	
I am happy to provide self-collected genital swabs (Exeter site only).	YES/NO	
I am happy to gift samples and data from the project to the Peninsula Research Bank in Exeter to be used for future research.	YES/NO	
I agree that information held by the NHS and in my medical records may be used to follow up on my future health status.	YES/NO	
I am happy to be contacted by my local research team about participating in other future studies.	YES/NO	

Participant Name		Signed		Date
				/ /
I confirm that I am on the delegation log for the TriMaster study to obtain consent. In my opinion the participant understands what this study involves and has capacity to take part.				
**Name of Person Obtaining Consent**		**Signed**		**Date**
				/ /
**Clinician Confirming Eligibility***		**Signed**		**Date**
				/ /

* Eligibility (eligible/non-eligible) confirmed as indicated on the screening CRF
When completed: 1 for participant; 1 for researcher site file; 1 to be kept in medical notes (front page only)

TriMaster Consent Form V3 08.05.2018 IRAS: 183044

Please stick consent barcode label below:

Visit 1 consent barcode	Visit 2 consent barcode
Visit 3 consent barcode	Visit 4 consent barcode

TriMaster Consent Form V3 08.05.2018 IRAS: 183044
