# Supplementary material for: TriMaster: randomised double-blind crossover study of a DPP4 inhibitor, SGLT2 inhibitor and thiazolidinedione as second-line or third-line therapy in patients with type 2 diabetes who have suboptimal glycaemic control on metformin treatment with or without a sulfonylurea—a MASTERMIND study protocol
Source: BMJ Open. 2020 Dec 21;10(12):e042784. doi: 10.1136/bmjopen-2020-042784 (PMC7754630; doi:10.1136/bmjopen-2020-042784)
Supplement: Supplementary data [file bmjopen-2020-042784supp003.pdf]

Data Category	Information
Primary registry and trial identifying number	ClinicalTrials.gov NCT02653209
Date of registration in primary registry	8 January 2016
Secondary identifying numbers	EudraCT 2015-002790-38; ISRCTN12039221; Sponsor 1603221; Funder MR/N00633X/1; HRA 16/SC/0147; IRAS 183044
Source(s) of monetary or material support	Medical Research Council, UK
Primary sponsor	Royal Devon and Exeter NHS Foundation Trust
Sponsor contact details	Alison Kerridge, Assistant R&D Manager;<br>Research and Development,<br>First Floor, Bowmoor House<br>Barrack Road,<br>Exeter<br>EX2 5DW<br>alison.kerridge@nhs.net; 01392 403055
Contact for public queries	Catherine Angwin, Trial Manager;<br>NIHR Exeter Clinical Research Facility<br>Room 02.15, RILD Building<br>Barrack Road<br>Exeter<br>EX2 5DW<br>Rde-tr.DiabetesDrugResponse@nhs.net; 01392 408180
Contact for scientific queries	Professor Andrew Hattersley FRCP FMedSci FRS, Chief Investigator;<br>University of Exeter Medical School<br>RILD Building<br>Barrack Road<br>Exeter<br>EX2 5DW<br>A.T.Hattersley@exeter.ac.uk; 01392 408260
Public title	TriMaster: Study of a DPP4 inhibitor, SGLT2 Inhibitor and Thiazolidinedione as Third Line Therapy in patients with Type 2 Diabetes.
Scientific title	TriMaster: Randomised Double-Blind Crossover Study of a DPP4 Inhibitor, SGLT2 Inhibitor and Thiazolidinedione as Third Line Therapy in Patients With Type 2 Diabetes Who Have Suboptimal Glycaemic Control on Dual Therapy with Metformin and a Sulphonylurea
Countries of recruitment	United Kingdom
Health condition(s) or problem(s) studied	Type 2 Diabetes
Intervention(s)	Drug: Sitagliptin (2x50mg as over-encapsulated hard shell to be taken orally, once a day); Drug: Canagliflozin (100mg as over-encapsulated hard shell to be taken orally, once a day); Drug: Pioglitazone (30mg as over-encapsulated hard shell to be taken orally, once a day)
	All participants receive all 3 interventional treatments for 16 weeks each in random order according to one of 6 possible treatment order (ABC, ACA, BAC, BCA, CAB, CBA) with no washout period
Key inclusion and exclusion criteria	Ages eligible for study: 30 – 80 years (inclusive); Sexes eligible for study: both; Accepts healthy volunteers: no

	Inclusion Criteria: Clinical diagnosis of Type 2 diabetes, Age ≥30 and ≤80, currently treated with two classes of oral glucose-lowering therapy that do not include a DPP4-inhibitor, a SGLT2 inhibitor or a thiazolidinedione, diabetes duration ≥12 months, no change in diabetes treatment (new treatments or dose change) within previous 3 months, HbA1c >58mmol/mol (7.5%) and ≤110mmol/mol (12.2%) – confirmed at screening visit, eGFR ≥60mls/min/1.73m^{2-} confirmed at screening visit, able and willing to give informed consent
	Exclusion Criteria: Changes in glucose-lowering therapy or dose within last 3 months, HbA1c ≤58mmol/mol (7.5%) or >110mmol/mol (12.2%), eGFR <60mls/min/1.73m^2, diabetes duration <12 months, ALT >2.5 x upper limit of the assay normal range or known liver disease, specifically >30µmol/L that is associated with other evidence of liver failure, insulin treated within the last 12 months, limb ischaemia shown by absence of both pulses in one or both feet, currently treated with corticosteroids, currently treated with rifampicin, gemfibrozil, phenytoin and carbamazepine, active infection (requiring antibiotics at present), foot ulcer requiring antibiotics within previous three months, recent (within 3 months) significant surgery or planned surgery (excluding minor procedures), acute cardiovascular episode (angina, myocardial infarction, stroke, transient ischemic episode) occurring within the previous 3 months, history of heart failure, current use of loop diuretic therapy (Furosemide or Bumetanide), history of bladder carcinoma, current/ongoing investigation for macroscopic haematuria, history of diabetic ketoacidosis, history of pancreatitis, pregnant, breastfeeding or planning a pregnancy over the study period, concurrent participation on another CTIMP (where IMP is currently being taken or without sufficient washout period), unable or unwilling to give informed consent
Study type	Interventional
	Allocation: Randomised; Intervention model: Crossover assignment; Masking: Double (Participant, Investigator)
	Primary Purpose: Treatment
	Phase 4
Date of first enrolment	1 November 2016
Target sample size	525 (reduced from 600)
Recruitment status	Active, not recruiting
Primary outcome(s)	On treatment HbA1c in obese patients (BMI>30kgm^{-2}) compared to non-obese patients (BMI<30kgm^{-2}), (time frame: 16 weeks); On treatment HbA1c in patients with

	an eGFR<90mls/min/1.73m^2 compared to patients with an eGFR>90mls/min/1.73m^2, (time frame: 16 weeks)
Key secondary outcomes	Patient preference [Time frame: 48-54 weeks (3x16 weeks of therapy)]; Prevalence of side effects [Time frame: 48-54 weeks (3x16 weeks of therapy)]; HbA1c on therapy against predefined test of gender heterogeneity (Time frame: 16 weeks)
Ethics Review	Status: Approved
	Date of Approval: 9 May 2016
	South Central – Oxford A Research Ethics Committee Nrescommittee.southcentral-oxforda@nhs.net
Completion Date	January 2021
Summary Results	N/A as study has not completed yet
