# Supplementary material for: TriMaster: randomised double-blind crossover study of a DPP4 inhibitor, SGLT2 inhibitor and thiazolidinedione as second-line or third-line therapy in patients with type 2 diabetes who have suboptimal glycaemic control on metformin treatment with or without a sulfonylurea—a MASTERMIND study protocol
Source: BMJ Open. 2020 Dec 21;10(12):e042784. doi: 10.1136/bmjopen-2020-042784 (PMC7754630; doi:10.1136/bmjopen-2020-042784)
Supplement: Author's manuscript [file bmjopen-2020-042784.draft_revisions.pdf]

BMJ Open is committed to open peer review. As part of this commitment we make the peer review history of every article we publish publicly available.

When an article is published we post the peer reviewers' comments and the authors' responses online. We also post the versions of the paper that were used during peer review. These are the versions that the peer review comments apply to.

The versions of the paper that follow are the versions that were submitted during the peer review process. They are not the versions of record or the final published versions. They should not be cited or distributed as the published version of this manuscript.

BMJ Open is an open access journal and the full, final, typeset and author-corrected version of record of the manuscript is available on our site with no access controls, subscription charges or pay-per-view fees (http://bmjopen.bmj.com).

If you have any questions on BMJ Open's open peer review process please email info.bmjopen@bmj.com

BMJ Open

TriMaster: Randomised Double-Blind Crossover study of a DPP4 inhibitor, SGLT2 inhibitor and thiazolidinedione as second or third line therapy in patients with Type 2 diabetes who have suboptimal glycaemic control on metformin treatment with or without a sulfonylurea – a MASTERMIND study protocol

Journal:	*BMJ Open*
Manuscript ID	bmjopen-2020-042784
Article Type:	Protocol
Date Submitted by the Author:	21-Jul-2020

Complete List of Authors:	Angwin, Catherine; University of Exeter, Medical School<br>Jenkinson, C; University of Exeter,<br>Jones, Angus; University of Exeter, The Institute of Biomedical & Clinical Science<br>Jennison, Christopher; University of Bath<br>Henley, W; University of Exeter<br>Farmer, Andrew; University of Oxford, Nuffield Department of Primary Care Health Sciencesd<br>Sattar, Naveed; University of Glasgow, Institute of Cardiovascular and Medical Sciences<br>Holman, Rury; University of Oxford Medical Sciences Division, Radcliffe Department of Medicine<br>Pearson, Ewan; University of Dundee<br>Shields, Beverley; University of Exeter<br>Hattersley, Andrew; University of Exeter

Keywords:	DIABETES & ENDOCRINOLOGY, Clinical trials < THERAPEUTICS, THERAPEUTICS

SCHOLARONE™
Manuscripts

BMJ

I, the Submitting Author has the right to grant and does grant on behalf of all authors of the Work (as defined in the below author licence), an exclusive licence and/or a non-exclusive licence for contributions from authors who are: i) UK Crown employees; ii) where BMJ has agreed a CC-BY licence shall apply, and/or iii) in accordance with the terms applicable for US Federal Government officers or employees acting as part of their official duties; on a worldwide, perpetual, irrevocable, royalty-free basis to BMJ Publishing Group Ltd ("BMJ") its licensees and where the relevant Journal is co-owned by BMJ to the co-owners of the Journal, to publish the Work in this journal and any other BMJ products and to exploit all rights, as set out in our licence.

The Submitting Author accepts and understands that any supply made under these terms is made by BMJ to the Submitting Author unless you are acting as an employee on behalf of your employer or a postgraduate student of an affiliated institution which is paying any applicable article publishing charge ("APC") for Open Access articles. Where the Submitting Author wishes to make the Work available on an Open Access basis (and intends to pay the relevant APC), the terms of reuse of such Open Access shall be governed by a Creative Commons licence – details of these licences and which Creative Commons licence will apply to this Work are set out in our licence referred to above.

Other than as permitted in any relevant BMJ Author's Self Archiving Policies, I confirm this Work has not been accepted for publication elsewhere, is not being considered for publication elsewhere and does not duplicate material already published. I confirm all authors consent to publication of this Work and authorise the granting of this licence.

For peer review only

TriMaster: Randomised Double-Blind Crossover study of a DPP4 inhibitor, SGLT2 inhibitor and thiazolidinedione as second or third line therapy in patients with Type 2 diabetes who have suboptimal glycaemic control on metformin treatment with or without a sulfonylurea – a MASTERMIND study protocol

Authors: CD Angwin[1,2], CE Jenkinson[1,2], AG Jones[1,3], C Jennison[4], WE Henley[5], AJ Farmer[6], N Sattar[8], RR Holman[5], ER Pearson[4], BM Shields[1,2]*, AT Hattersley[1,3]* for the MASTERMIND consortium

these authors contributed equally

Affiliations:

[1]Institute of Biomedical & Clinical Sciences, University of Exeter Medical School, Exeter, UK

[2]NIHR Exeter Clinical Research Facility, Royal Devon and Exeter NHS Foundation Trust, Exeter, UK

[3]Diabetes and Endocrinology, Royal Devon and Exeter Hospital NHS Foundation Trust, Exeter, UK

[4]Department of Mathematical Sciences, University of Bath, Bath, UK [4]Division of Population Health and Genomics, Ninewells Hospital and School of Medicine, University of Dundee, Dundee, Scotland, UK

[5]Institute of Health Research, University of Exeter Medical School, Exeter, UK

[6]Nuffield Department of Primary Care Health Sciences, University of Oxford, Oxford, UK

[7]Institute of Cardiovascular and Medical Sciences, University of Glasgow, Glasgow, UK

[8]Radcliffe Department of Medicine Diabetes Trial Unit (OCDEM), University of Oxford, Oxford, Oxfordshire, UK

Corresponding authors:

Prof Andrew Hattersley A.T.Hattersley@exeter.ac.uk

Dr Beverley Shields B.Shields@exeter.ac.uk

Word count: 4285 – excluding abstract, references, figures and tables

For peer review only - http://bmjopen.bmj.com/site/about/guidelines.xhtml

ABSTRACT

Introduction

Pharmaceutical treatment options for patients with type 2 diabetes (T2DM) have increased to include multiple classes of oral glucose-lowering agents but without accompanying guidance on which of these may most benefit individual patients. Clinicians lack information for treatment intensification after first-line metformin therapy. Stratifying patients by simple clinical characteristics may improve care by targeting treatment options to those in whom they are most effective. This academically-designed and run three-way crossover trial aims to test a stratification approach using three standard oral glucose-lowering agents.

Methods and analysis

TriMaster is a randomised, double-blind, crossover trial taking place at up to 25 clinical sites across England, Scotland and Wales. 520 patients with T2DM treated with either metformin alone, or metformin and a sulfonylurea who have HbA_{1c} >58mmol/mol will be randomised to receive 16 weeks each of a DPP4 inhibitor, SGLT2 inhibitor and thiazolidinedione in random order. Participants will be assessed at the end of each treatment period, providing clinical and biochemical data, and their experience of side effects. Participant preference will be assessed on completion of all 3 treatments. The primary endpoint is HbA_{1c} after 4 months of therapy (allowing a range of 12-18 weeks for analysis. Secondary endpoints include participant-reported preference between the three treatments, tolerability and prevalence of side effects.

Ethical approval: This study was approved by NHS Health Research Authority (HRA) Research Ethics Committee South Central – Oxford A, study 16/SC/0147. Written informed consent will be obtained from all participants. Results will be submitted to a peer-reviewed journal and presented at relevant scientific meetings. A lay summary of results will be made available to all participants.

Trial registration number: ISRCTN: 12039221. EudraCT: 2015-002790-38. ClinicalTrials.gov: NCT02653209.

For peer review only - http://bmjopen.bmj.com/site/about/guidelines.xhtml

ARTICLE SUMMARY

Strengths and limitations of this study:

- This is the first blinded three-way crossover of glucose-lowering therapies in T2DM, allowing comparison of short term treatment response and side effects across 3 agents within the same individuals
- This study design enables assessment of stratification allowing for within-person variation in response
- This will be the first study to assess patient preference for choosing between 3 glucose-lowering therapies
- A limitation is that only short-term glycaemic response and side effects can be assessed in a study of this design

BACKGROUND AND RATIONALE

In recent years the choice of therapies designed to lower glucose in patients with T2DM has increased(1) but there remains limited information as to which patients may respond well, moderately, or poorly to any of the treatment options(2, 3). Treatment intensification is recommended in a step-wise approach, with guidelines usually including a number of different agents after metformin in those without established cardiac or renal disease(2, 4).

T2DM is a heterogenous condition and response to glucose-lowering therapy appears to vary substantially between individuals. Therefore identification of subgroups of patients who respond well or poorly to a specific therapy, or with an altered risk of treatment-specific side effects, could improve targetting of treatment. This stratified approach to therapy is most likely to be successful if based on clinical characteristics and biomarkers that are readiliy available in routine clinical care: T2DM is common, most therapies are relatively inexpensive and most management is undertaken in primary care, therefore stratification based on expensive biomakers, or those with limited availability, is unlikely to be widely adopted.

A number of previous studies have shown simple clinical characteristics and biomarkers are associated with variation in glycaemic response for individual therapies(5-8). However, to be most useful for stratification a marker needs to predict differential response between therapies(9). Work by the MASTERMIND consortium using routine and trial data has strengthened the evidence that clinical features are associated with differential glycaemic response to DPP4-inhibitors, SGLT2-inhibitors and thiazolidinediones (10-12). Analysis of data from the UK Clinical Practice Research

For peer review only - http://bmjopen.bmj.com/site/about/guidelines.xhtml

Datalink (CPRD) and ADOPT trial showed that sex and BMI above and below 30 were associated with differential glycaemia response between sulfonylureas and thiazolidinediones (10). In addition, individuals within a normal estimated glomerular filtration rate (eGFR) range, with a higher eGFR show a better glycaemia response to Sodium-glucose co-transporter-2 (SGLT2) inhibitors whilst individuals with a lower eGFR may have a better glycaemic response when taking a Dipeptidyl peptidase-4 (DPP4) inhibitor (Janssen, personal communication from MASTERMIND industry group, 2014). The features identified sex, obesity and renal function, are routinely measured at low cost, meaning potential stratification using these characteristics could be easily implemented in clinical practice.

TriMaster aims to test potential glycaemic therapy stratification in T2DM using response to three standard glucose-lowering agents. It will determine whether subgroups defined by routinely measured features respond to a greater or lesser degree (with regard to glycaemic change) to DPP4-inhibitors, SGLT2-inhibitors and thiazolidinediones, and provide a resource for further investigation of stratification between these therapies in the future.

These therapies were selected on the basis of differential response seen in pilot studies, and the choice of available oral third-line therapies at the start of the study.

Hypotheses:

The trial is designed with the following hypotheses:

1. Patients with insulin resistance, characterised clinically by a raised BMI (>30kg/m^2), compared to non-obese patients will: (a) respond well to pioglitazone, a thiazolidinedione that works as an insulin sensitiser(13); (b) respond less well to sitagliptin, a DPP4-inhibitor which works through stimulating endogenous insulin secretion post-prandially(14).

2. Patients with modestly reduced eGFR (60-90 mls/min/1.73m^2), compared to those with eGFR >90 mls/min/1.73m^{2,} will: (a) respond less well to canagliflozin, an SGLT2 inhibitor, which works through inhibiting the active reabsorption of glucose in the proximal tube(15), as the reduced eGFR will reduce the glucose-lowering efficacy; (b) respond well to sitagliptin, a DPP4 inhibitor that is renally cleared, as the reduced eGFR will increase plasma DPP4 inhibitor concentrations.

PRIMARY OBJECTIVES

To test two hypotheses of drug response stratification based on drug mechanism of action and pharmacokinetics to answer the following clinical questions:

For peer review only - http://bmjopen.bmj.com/site/about/guidelines.xhtml

For peer review only

1. Do obese patients (BMI >30kg/m^2), compared to non-obese patients, achieve a lower HbA_{1c} when assigned to pioglitazone rather than sitagliptin?
2. Do patients with an eGFR 60-90 mls/min/1.73m^2 achieve a lower HbA_{1c}, compared to patients with an eGFR >90 mls/min/1.73m^2, when assigned to sitagliptin rather than canagliflozin?

SECONDARY OBJECTIVES

The design of the study provides people with T2DM the unusual opportunity to try a panel of three available glucose-lowering therapies and to express a preference based on their experience of each. The study's secondary objectives are to determine:

1. Patient treatment preference within hypothesised strata and overall.
2. Prevalence of side effects within hypothesised strata and for specific drugs, to include: weight gain, hypoglycaemia, oedema, genital tract infection and discontinuation of therapy.
3. Predefined test of sex heterogeneity with pilot data suggesting females are more likely to show an improved response relative to males for pioglitazone.
4. Tolerability of treatments within hypothesised strata and overall.

METHODS AND ANALYSIS

We have used the SPIRIT reporting guidelines in the design of the protocol and preparation of this paper(16).

Overview of trial design

TriMaster is a phase four, academically-designed and run, multi-centre, randomised, double-blind, 12-month crossover trial of a DPP4 inhibitor (sitagliptin), thiazolidinedione (pioglitazone) and SGLT2 inhibitor (canagliflozin) as a second or third line therapy in patients with T2DM who have suboptimal glycaemic control on metformin alone or metformin and sulfonylurea (Figure 1 – TriMaster Consort diagram). The three-way crossover will be undertaken as an efficient, faster and more cost-effective approach to address both hypotheses, requiring fewer participants than performing two 2-way cross over studies.

520 participants with T2DM will be recruited, aged 30-80 years on stable doses of metformin alone or metformin and a sulfonylurea with glycated haemoglobin (HbA_{1c}) >58mmol/mol (>7.5%) and ≤110mmol/mol (≤12.2%). Each participant will attend one screening and, if eligible, five research

For peer review only - http://bmjopen.bmj.com/site/about/guidelines.xhtml

visits over a 12-month period (50-60 weeks max/min visit windows). They will receive the three blinded second and third-line oral therapies in random order for 16-18 weeks each, with no washout period between therapies. Participant feedback from pilot studies found repeated washout periods increased rates of withdrawal due to poorly tolerated hyperglycaemia. Once stopped, none of the three glucose-lowering agents used in this study have a continuing glucose-lowering effect beyond four weeks (all three drugs have half-lives between 7 and 14 hours so their effects should be negligible after a week)(17-19) and HbA_{1c} measurement reflects glycaemia over the preceding eight to twelve-week period. HbA_{1c} measurements taken after 16 weeks will therefore allow a 4-week 'wash-in' period and effectively reflect response to each treatment period.

Participants will each act as their own control, and on completion of all three treatments will be asked to rank the treatments taken in order of preference. Eligible participants will be recruited at 20-25 UK sites; the trial is sponsored by the Royal Devon and Exeter NHS Foundation Trust and hosted at the National Institute for Health Research (NIHR) Exeter Clinical Research Facility. A full list of recruiting sites will be available via the ISRCTN registration.

Eligibility criteria

All potential participants will undergo a formal screening visit to assess and confirm eligibility as listed in Table 1.

Table 1. Eligibility Criteria

**Inclusion criteria**	**Exclusion criteria**
• Clinical diagnosis of T2DM<br>• Age ≥30 and ≤80 years<br>• Currently treated with one or two classes of oral glucose-lowering therapy (given either as separate or combined medications), that do not include a DPP4-inhibitor, a SGLT2-inhibitor or a thiazolidinedione<br>• Diabetes duration ≥12 months	• Changes in glucose-lowering therapy or dose within last 3 months<br>• ALT >2.5 x upper limit of the assay normal range (ULN) or known liver disease, specifically bilirubin >30 µmol/L that is associated with other evidence of liver failure<br>• Insulin treated within the last 12 months<br>• Treated with study dugs within the last 3 months

For peer review only - http://bmjopen.bmj.com/site/about/guidelines.xhtml

• No change in diabetes treatment (new treatments or dose change) within previous 3 months<br>• HbA_{1c} > 58mmol/mol (>7.5%) and ≤110mmol/mol (≤12.2%)<br>• eGFR ≥ 60mls/min/1.73m²<br>• Able and willing to give informed consent	• Limb ischaemia shown by absence of both pulses in one or both feet<br>• Currently treated with corticosteroids<br>• Currently treated with rifampicin, gemfibrozil, phenytoin and carbamazepine<br>• Active infection (any infection requiring antibiotics)<br>• Foot ulcer requiring antibiotics within previous three months<br>• Recent (within 3 months) significant surgery or planned surgery (excluding minor procedures)<br>• Acute cardiovascular episode (angina, myocardial infarction, stroke, transient ischemic episode) occurring within the previous 3 months<br>• History of heart failure<br>• Current use of loop diuretic therapy (Furosemide or Bumetanide)<br>• History of bladder carcinoma<br>• Current/ongoing investigation for macroscopic haematuria<br>• History of diabetic ketoacidosis<br>• History of pancreatitis<br>• Pregnant, breastfeeding or planning a pregnancy over the study period<br>• Concurrent participation on another Clinical Trial of an Investigational Medicinal Product (CTIMP) where the IMP is currently being taken, or without sufficient washout period (five times the longest half-life of the study IMPs) and without consultation with the CTIMP research team

For peer review only

For peer review only - http://bmjopen.bmj.com/site/about/guidelines.xhtml

	• Unable or unwilling to give informed consent<br>• Females of childbearing potential must be willing to use an effective method of contraception from the time consent is signed until seven days after treatment discontinuation. A negative pregnancy test is required within seven days prior to treatment initiation and will be required for continuation at each study visit

Outcome measures

In line with WHO guidelines, response to therapy will be assessed by measurement of glycated haemoglobin (HbA_{1c})(20). The primary outcome is the HbA_{1c} value achieved after each 16-week treatment period. Should a participant be unable to complete a full 16-week treatment period, HbA_{1c} will be measured and included in the main analysis if the participant has taken the study drug for at least 12 weeks.

Secondary outcomes will be participant-reported preference between the three treatments, tolerability of the three treatments and prevalence of side effects. In addition, we will explore sex differences in response to the three drugs.

Participant willingness to continue a drug long term will be recorded at the end of each treatment arm. Treatment preference, taken as a ranking of the three study drugs will be recorded at study completion. To inform this decision, in addition to their experience on each drug, clinical information including HbA_{1c} measurements and weight change will be fed-back to each participant. Frequency and severity of side effects will be recorded throughout the study alongside the Diabetes Treatment Satisfaction Questionnaire (DTSQ) to allow a formal validated assessment of participant satisfaction.

HbA_{1c} assessment during the study will be performed in local NHS laboratories to ensure results are available for screening, and to inform final patient preference. Central laboratory analysis will be undertaken at the Exeter Clinical Laboratory at the Royal Devon & Exeter NHS Foundation Trust for all other non-safety sample analysis. All analyses are routine biochemistry tests available in the NHS

For peer review only

test repertoire. All assays are CE marked, fully validated and accredited by the United Kingdom Accreditation Service (UKAS).

Sample size

The primary outcome is HbA_{1c} at the end of each treatment period.

This trial aims to test whether participants in a particular strata (S) respond differently to drug A and drug B compared with patients not in the strata (N). The primary outcome is the HbA_{1c} measurement after 4 months of each drug.

Table 2: Differences in response between two drugs and two strata in the crossover trial

Patient group	Drug A	Drug B	Difference
In strata (S)	$HbA1c_{SA}$	$HbA1c_{SB}$	$HbA1c_{SA}$ - $HbA1c_{SB}$
Not in strata (N)	$HbA1c_{NA}$	$HbA1c_{NB}$	$HbA1c_{NA}$ - $HbA1c_{NB}$

The null hypothesis is that the difference in achieved HbA_{1c} for the two drugs will be similar for the two groups of participants (i.e. $HbA1c_{SA}$-$HbA1c_{SB}$ = $HbA1c_{NA}$-$HbA1c_{NB}$ in Table 2).

In a crossover trial of metformin versus repaglinide the standard deviation (SD) of change in HbA_{1c} on the two different therapies was 8.7mmol/mol(21). Analysis of Clinical Practice Research Datalink (CPRD) showed obese patients respond better to TZDs and non-obese patients respond better to DPP4is, with an overall difference in response between strata of 3.1mmol/mol (equivalent to 0.36SDs). Similarly, higher eGFR >90ml/min/1.73m² is associated with a better HbA_{1c} response to SGLT2i, whilst patients with an eGFR 60-90ml/min/1.73m² had a lower response to the SGLT2i and higher response to DPP4i with an overall difference in response between strata of 3.0mmol/mol (equivalent to 0.35SDs) (Janssen, personal communication, 2014).

Using 90% power, alpha=0.05, to detect a difference of 0.35SDs we require 172 participants in each stratum, 344 in total. To allow for the possibility of unequal numbers in each stratum, the sample size has been increased to 358, assuming a 60:40 split (T2DM population CPRD 52:48 for both strata; a conservative withdrawal rate of 15% increases the study sample size to 422. To allow for participants excluded from primary analysis due to less than 12 weeks on one or more study drugs (estimated at 19%), we will increase the total sample size for the study to 520.

Investigational Medicinal Product (IMP)

For peer review only - http://bmjopen.bmj.com/site/about/guidelines.xhtml

Trial interventions were chosen in line with UK NICE guidelines for first and second intensification of drug treatment in patients with T2DM(2). The three drugs will be provided to participants in a blinded format and randomised order at the starting dose indicated in the British National Formulary (BNF); sitagliptin 100mg, canagliflozin 100mg, pioglitazone 30mg.

IMPs will be supplied directly to recruiting site pharmacies by Tayside Pharmaceuticals, Dundee, UK. Tablets will be over-encapsulated in a hard gelatin capsule so that the IMPs are near identical in size and colour, and packed into bottles and distributed to each recruitment site (see randomisation section for further details).

Participants will be instructed to take one capsule, once daily, alongside their existing diabetes treatment and usual medications. They will be provided a Drug Information Sheet in place of a standard Summary of Product Characteristics stating the expected side effects of all three treatments. To allow feasible visit windows and prevent participants running out of IMP, each IMP bottle will contain 126 capsules, the equivalent of 18 weeks' medication.

Where a participant is unable to tolerate a therapy, they will move to the next IMP in their randomised order, providing they remain clinically safe to continue in the study. Dose modification, reduction or delay will not be permitted due to the blinded nature of the trial. Participants will be asked to return the IMP bottle and all unused capsules; research staff will perform a capsule count for adherence and accountability purposes.

Randomisation, allocation and blinding

The study has six treatment sequence permutations: ABC, ACB, BAC, BCA, CAB and CBA; participants will be randomly allocated to one of the six sequences when confirmed as eligible in the study database. The study is double-blind and all clinical, participant and laboratory assessments will be made prior to database lock, final analysis, and unblinding of the drug order.

A block randomisation list (block size 12) will be created using Statsdirect by the Trial Statistician and the randomisation seed recorded. The randomisation list will be provided to the study database team who will randomly allocate blocks of 12 to each of the recruitment sites (to ensure balance between the 6 treatment orders at each site), with the allocation remaining blinded to the rest of the study team. The IMP supplier will provide each site with blocks of 12 drugs (4 of each of the 3 study drugs) with the 12 bottle IDs labelled in random order to avoid the drug type being easily

For peer review only - http://bmjopen.bmj.com/site/about/guidelines.xhtml

identifiable. The bottle IDs and contents assigned to each recruitment site will be recorded in the study database and accessible only to the database team.

To ensure allocation concealment, randomisation will be centralised via the study database. Eligibility will be confirmed by research teams and the participant randomised to a blinded treatment order, allocated by the study database. Prior to research visits one, two and three, the database will allocate the next available IMP bottle of the correct drug type held at that site, according to this treatment order. Study prescriptions detailing the allocated bottle ID will be processed and dispensed by the site clinical trials pharmacy.

Participants will not be recruited against specific strata; BMI and eGFR defined stratum will be monitored as recruitment and randomisation progresses. Data on the distribution will be provided to the Data Monitoring Committee and if enrolment is unevenly distributed to an extent that the study hypotheses cannot be robustly tested, the relevant stratum may be ‘switched off’ by the Data Programmer to prevent further randomisation into the relevant strata.

Participants who withdraw before randomisation will be replaced. However, once randomised, their data will be included in analysis. To maintain data quality and trial integrity, unblinding via code breaks will occur only in exceptional circumstances where knowledge of the IMP is deemed essential for the correct clinical management of the participant, a medical emergency where someone other than the participant has taken the IMP, or where this information is needed to establish expectedness of a potential Suspected Unexpected Serious Adverse Reaction (SUSAR). The emergency code break table is available electronically on the study database requiring multiple confirmation steps to avoid accidental unblinding, and on paper in the central coordinating centre. In the event a code break is required this will be done by a member of staff independent of the main trial team. A study involvement card with study ID, IMP details and contact information for local and central emergency unblinding will be provided to all participants.

Study visits and procedures

Figure 2 illustrates the schedule of assessments.

Identification and recruitment

Potential participants will be identified through primary and secondary care, research databases and direct clinician referral, provided with an information sheet, and invited to attend a screening visit.

For peer review only - http://bmjopen.bmj.com/site/about/guidelines.xhtml

Following informed consent by trained and delegated research staff, clinical characteristics (height, weight, waist and hip circumference), medical history, concomitant medication details and non-fasting blood samples will be collected to confirm eligibility. Eligible participants will be randomised into the trial, assigned a unique Study ID and allocated a drug order.

Baseline visit

Within two weeks of screening, participants will attend a fasting baseline research visit. Baseline physiological data will be collected, along with self-reported compliance to existing diabetes medication. Participant's personal priorities in choosing between treatment options and experience of side effects on current treatment will also be recorded. Participants will have underlying pathophysiology assessed in a two-hour mixed-meal tolerance test (MMTT) using a standard meal drink (Fortisip). The meal test will be undertaken using 250ml of Nutricia Fortisip or 160ml of Nutricia Fortisip Compact. Equivalent products may be used where Fortisip cannot be tolerated. Blood and urine samples will be collected for analysis and future biomarker discovery at baseline, and then at 30-minute intervals (0, 30, 60, 90 and 120 minutes) following the meal drink. Participants at the central Exeter site will also be invited to provide a self-collected genital swab sample to identify development of sub-clinical colonisation of candida or bacteria.

Subsequent research visits will take place after 16-18 weeks of study treatment. However, patients will be offered the opportunity to stop a treatment early and move onto the next treatment period if they are unable to tolerate the therapy. Visits will repeat the baseline physiological measurements with samples collected at a single time point. Participants will provide fasting blood samples for immediate measurement of HbA_{1c}, and subsequent assessment to include fasting glucose, c-peptide, insulin, glycosylated albumin, creatinine, lipid profile and drug levels. Weight, blood pressure, adherence and data about patient experience will also be collected, including perceived side effects, preparedness to remain on the drug long term, and health-related quality of life. Where collected at baseline, subsequent genital swabs will be repeated at study visits two to four.

Case Report Forms (CRFs) will be completed at recruiting centres and securely transferred to the central team via nhs.net email. OpenText TeleForm will be used for data capture and transfer to the study database. Identifiable data will be securely stored at recruiting centres, research data transferred to Exeter will be accessed only by delegated members of the research team.

Questionnaires - Participant preference

For peer review only - http://bmjopen.bmj.com/site/about/guidelines.xhtml

On completion of the third study drug, participants will be provided with a summary of their previous assessments of each therapy. At a final study visit, participants will first rank the study drugs based solely on their own experience on treatment. HbA_{1c} and weight data for each drug period will then be provided by the research team, and a repeat ranking recorded. This summary assessment was developed with the TriMaster Patient Involvement Group and the Peninsula Research Bank Lay Committee members. Endpoints of willingness to remain on study drug long term, and impact on daily life were identified as the best representations to capture participant preference for the study.

A final version of drug preference and clinical data will be provided to the participant and their clinician. This document, provided directly by the Exeter CTU team to ensure research teams remain blinded throughout, will contain details of the unblinded study drugs A, B and C to inform future treatment choices. All study procedures will occur within the 50-60 week trial period but permission will be requested to contact participants after primary analysis is complete to assess future treatment choice.

Participants will also complete the Diabetes Treatment Satisfaction Questionnaire (DTSQ) at baseline and the change version (DTSQc) after each treatment period to collect validated satisfaction scores.

Statistical analysis

Analyses and reporting will follow CONSORT guidance for randomised crossover trials(22). This study is not designed to test drug efficacy but the effectiveness of stratification. Therefore, only patients completing at least 12 weeks on therapy (sufficient to allow HbA_{1c} to reflect glycaemia control on the drug) will be included in primary analysis. In addition, we will perform a secondary analysis of tolerability examining whether the proportion of participants completing at least 12 weeks differs for each drug, both within strata and overall.

Prior to main analysis, we will determine whether there is any evidence of carryover or period effects. Any carryover effect identified will be reported but not adjusted for in subsequent analysis. Period effects will be reported and adjusted for. We do not anticipate treatment effect carryover and have designed the study to limit potential carryover (as far as possible). Any period effect in the maximum 8 months between on treatment HbA_{1c}s is likely to be minimal as mean progression is 1.0 mmol/mol/year (personal communication E Pearson, data from GoDARTs population data).

For peer review only - http://bmjopen.bmj.com/site/about/guidelines.xhtml

There will be two primary analyses, one for each of the study hypotheses. For each hypothesis the primary analysis will be to assess whether the difference in achieved HbA_{1c} measurements for the two drugs is similar for the two groups of participants. The two hypotheses will be tested separately using linear mixed effects models to compare the strata on the two drugs of interest, with a random effects term for the participant. The key contrast of interest is the drug*strata interaction, where the strata is either obesity group or eGFR group. To determine whether there is a difference between drug classes in terms of the overall achieved HbA_{1c} after 4 months on each of the drugs, we will fit an additional model. Drug will be a factor and coded as a dummy variable as the comparison will be across 3 rather than 2 drug classes. Least square means will be extracted for the 3 drugs. Similar analysis will be carried out with weight after 4 months as the outcome.

In addition, we will examine the distribution of side effects reported across each of the 3 drugs. However, given the total numbers reporting each individual side effect will likely be small, we anticipate this will largely be descriptive, examining proportion of side effects observed with each drug.

For analysis of patient preference, we will only analyse the dataset where the participants have tried all 3 drugs. The mean rank for each drug will be calculated and tested against the null hypothesis that there is not a preferred drug and therefore the expected value of the rank for a given drug will be 2. Further investigation of patient preference will be exploratory.

Reasons for missing data will be documented and the baseline characteristics of those with and without missing data compared.

Monitoring

Due to the nature of standard diabetes treatments, it is expected that participants will experience some mild adverse events or reactions. These will be recorded at research visits and reported on a study-wide basis to the sponsor and Data Monitoring Committee at regular intervals.

Serious Adverse Reactions where the IMP is assessed as having possible, probable or definite causality will be unblinded to enable full evaluation of expectedness in the context of the relevant safety profile. Independent auditing of the trial will be arranged by the sponsor, in addition to sponsor review.

For peer review only

Oversight committees

The study will be run by a Trial Management Group (TMG) with oversight from an independent Trial Steering Committee (TSC) and Data Monitoring Committee (DMC). These committees will meet regularly to monitor the scientific integrity and safety of the trial and provide independent advice. To ensure the safety of participants, the DMC will review unblinded safety data.

Ethics and Dissemination

This study has been reviewed and received ethics approval from the NHS Health Research Authority (HRA) Research Ethics Committee South Central – Oxford A, study 16/SC/0147. The Clinical Trial Application was reviewed and approved by the UK Medicines and Healthcare products Regulatory Agency (MHRA) under EudraCT reference: 2015-002790-38. All substantial and non-substantial amendments have received approval from HRA, REC and MHRA before implementation. The protocol has been registered with ClinicalTrials.gov (NCT02653209) and ISRCTN (12039221).

All participants will be provided verbal and written information about the study prior to providing written informed consent and will be free to withdrawn at any time without affecting current or future clinical treatment.

Changes to protocol

The study was first registered with Clinicaltrials.gov (NCT02653209) on 12.01.2016 and ISRCTN (ISRCTN12039221) on 02.11.2016. Table 3 shows the approved protocol amendments since this date.

Table 3. Protocol Amendments

**Protocol Amendments**	
**SA1**<br>v3 06.07.16	Amendment to randomisation process to allocate individual bottles rather than 'packs' of 3 bottles to allow for shorter expiry dates, and clarification of safety reporting procedures
**SA4**<br>v4 20.03.17	Amendment to exclusion criteria to allow patients who have previously tried the study drugs to be included, as long as this has not been in the previous 3 months. The original criteria was unnecessarily strict and did not reflect real-world prescribing habits. The amendment also removed the blanket exclusion for patients in concurrent clinical trials, providing sufficient washout period between IMPs.
**SA6**<br>v5 01.08.17	Amendment to eligibility criteria to include patients taking metformin-only, or metformin and a sulfonylurea. This was adjusted due to the change in guidelines and prescribing trends leading to decline in use of sulfonylureas. At the time of study design sulfonylureas were the most commonly prescribed second line therapy in the UK. Subsequent decline in their use in favour of DPP4-inhibitors and SGLT2 inhibitors (2), resulted in the inclusion of patients currently treated with either metformin and sulfonylureas or metformin only. We will perform a sensitivity analysis to determine if the difference in study "epoch" (before/after this amendment) has any impact on the main study outcomes.<br>Altered exclusion criteria also added 'limb ischaemia' due to updated safety information for Canagliflozin, and an upper limit of HbA1c >110mmol/mol.

For peer review only - http://bmjopen.bmj.com/site/about/guidelines.xhtml

**SA9** v6 15.05.18	Amendment to sample size due to over-cautious sample calculations (alpha changed to 0.05), extension to recruitment period due to delays in regulatory approvals at study set-up and slow early recruitment, and additional secondary analysis included on the advice of the Data Monitoring Committee.
**SA10** v7 22.02.19	Amendment to study analysis plan. Following advice from the Trial Steering Committee statistician, the protocol was amended to analyse only those completing at least 12 weeks on therapy, as this will determine whether the strata result in differences in response (we cannot adequately measure glycaemic response by HbA_{1c} if the patient has been on the drug for less than 12 weeks). A separate analysis will be performed to determine whether the strata influence tolerability by assessing whether the proportion completing at least 12 weeks on therapy differs by drug and strata.
**SA12** v8 20.03.20	Amendment to ensure ongoing participant safety and study integrity during Covid-19 pandemic. Urgent safety measures included (i) extension of visit windows to 14-18 weeks to allow greater flexibility for participants who are unwell/isolating, (ii) provision for remote visits with sample collection outside the usual research setting, (iii) ensuring participants remained on study therapy when only a remote visit is possible, by allowing an additional 'continuation' bottle of the same IMP to be issued, or when no other option, transfer to the next IMP without collection of blood samples.

Dissemination

Data and results related to protocol-derived outcomes will be published in peer-reviewed journals by the Chief Investigator on behalf of the MASTERMIND consortium and presented at scientific meetings. Anonymous trial data will be shared within the MASTERMIND consortium and after publication of results, data will be securely deposited in Exeter's institutional repository and made available on request via the consortium's data access group. A lay summary will be provided to all study participants and made available on the study website, and public registries.

Patient and Public Involvement

Patients were involved in the design and conduct of this study. Following pilot studies, the TriMaster Patient Involvement Group provided feasibility feedback on the study design and the outcome measures used to record patient preference and experience of the study drugs. This group and the Peninsula Research Bank Lay Committee members assisted in the design of patient-facing documents, including consent forms, study and drug information sheets and data collection forms to assess patient preference.

Funding statement:

This work is part of the MASTERMIND (MRC APBI Stratification and Extreme Response Mechanism IN Diabetes) consortium and is supported by the UK Medical Research Council study grant number MR/N00633X/1. The funder and sponsor had no role in study design, and will not have a role in

For peer review only - http://bmjopen.bmj.com/site/about/guidelines.xhtml

collection, management, analysis and interpretation of data, or decision to submit results for publication. The TriMaster trial is supported by the National Institute for Health Research (NIHR) Exeter Clinical Research Facility. The views expressed are those of the authors and not necessarily those of the MRC, NHS, the NIHR or the Department of Health. ERP holds a Wellcome Trust New Investigator Award (102820/Z/13/Z). AGJ is supported by an NIHR Clinician Scientist award (CS-2015-15-018). NS is supported by a BHF Centre of Excellence Award (RE/18/6/34217). RRH is an Emeritus National Institute for Health Research Senior Investigator. ATH is a Wellcome Senior Investigator (098395/Z/12/Z) and a Senior Investigator at the NIHR. ATH, AGJ, BS and CA are supported by the NIHR Exeter Clinical Research Facility. AF is an NIHR Senior Investigator and receives support from NIHR Oxford Biomedical Research Centre

Authors' contributions: state how each author was involved in writing the protocol:

ATH, BMS, AGJ, ERP, RRH, CJ, AJF, NS, WEH and CDA were involved in conception and trial design. CDA, CEJ, BMS and AGJ were involved in drafting of the article. ATH, BMS, AGJ, ERP, RRH, CEJ, AJF, CJ, WEH and NS were involved in critical revision of the article. All the authors were involved in final approval of the article.

Competing interest statement

ERP has received Honoraria from Lilly. NS has consulted for Amgen, Astrazeneca, Boehringer Ingelheim, Eli-Lilly, Napp, NovoNordisk, Sanofi and Pfizer and received grant funding from Boehringer Ingelheim. RRH reports research support from AstraZeneca, Bayer and Merck Sharp & Dohme, and personal fees from Bayer, Intarcia, Merck Sharp & Dohme, Novartis and Novo Nordisk outside the submitted work. CJ has consulted for AstraZeneca, Boehringer Ingelheim, NovoNordisk and Sanofi. WEH has received grant funding from IQVIA and travel funds from Eisai.

For peer review only - http://bmjopen.bmj.com/site/about/guidelines.xhtml

Figures:

1. TriMaster Consort Diagram
2. TriMaster Schedule of Assessment
3. TriMaster SPIRIT Checklist

For peer review only

For peer review only - http://bmjopen.bmj.com/site/about/guidelines.xhtml

REFERENCES

1. Qaseem A, Barry MJ, Humphrey LL, Forciea M, for the Clinical Guidelines Committee of the American College of P. Oral pharmacologic treatment of type 2 diabetes mellitus: A clinical practice guideline update from the american college of physicians. Annals of Internal Medicine. 2017;166(4):279-90.
2. Dennis JM, Henley WE, McGovern AP, Farmer AJ, Sattar N, Holman RR, et al. Time trends in prescribing of type 2 diabetes drugs, glycaemic response and risk factors: A retrospective analysis of primary care data, 2010-2017. Diabetes, obesity & metabolism. 2019;21(7):1576-84.
3. Pearson ER. Personalized medicine in diabetes: the role of 'omics' and biomarkers. Diabetic Medicine. 2016;33(6):712-7.
4. Davies MJ, D'Alessio DA, Fradkin J, Kernan WN, Mathieu C, Mingrone G, et al. Management of hyperglycaemia in type 2 diabetes, 2018. A consensus report by the American Diabetes Association (ADA) and the European Association for the Study of Diabetes (EASD). Diabetologia. 2018;61(12):2461-98.
5. Jones AG, McDonald TJ, Shields BM, Hill AV, Hyde CJ, Knight BA, et al. Markers of beta-Cell Failure Predict Poor Glycemic Response to GLP-1 Receptor Agonist Therapy in Type 2 Diabetes. Diabetes care. 2016;39(2):250-7.
6. DeFronzo RA, Stonehouse AH, Han J, Wintle ME. Relationship of baseline HbA1c and efficacy of current glucose-lowering therapies: a meta-analysis of randomized clinical trials. Diabetic Medicine. 2010;27(3):309-17.
7. Thong KY, McDonald TJ, Hattersley AT, Blann AD, Ramtoola S, Duncan C, et al. The association between postprandial urinary C-peptide creatinine ratio and the treatment response to liraglutide: a multi-centre observational study. Diabetic Medicine. 2014;31(4):403-11.
8. Bihan H, Ng WL, Magliano DJ, Shaw JE. Predictors of efficacy of GLP-1 agonists and DPP-4 inhibitors: A systematic review. Diabetes Research and Clinical Practice. 2016;121:27-34.
9. Dennis JM. Precision medicine in type 2 diabetes: Using individualised prediction models to optimise selection of treatment. Diabetes. 2020;(in press).
10. Dennis JM, Henley WE, Weedon MN, Lonergan M, Rodgers LR, Jones AG, et al. Sex and BMI Alter the Benefits and Risks of Sulfonylureas and Thiazolidinediones in Type 2 Diabetes: A Framework for Evaluating Stratification Using Routine Clinical and Individual Trial Data. Diabetes care. 2018;41(9):1844-53.
11. Dennis JM, Shields BM, Hill AV, Knight BA, McDonald TJ, Rodgers LR, et al. Precision Medicine in Type 2 Diabetes: Clinical Markers of Insulin Resistance Are Associated With Altered Short- and Long-term Glycemic Response to DPP-4 Inhibitor Therapy. Diabetes care. 2018;41(4):705-12.
12. Rodgers L, ER. P, Hammersley S, Angwin C, McDonald TJ, Shields BM, et al. Patients with a high fasting glucose respond better to sulphonylureas than dipeptidylpeptidase IV (DPP-IV) inhibitors: a MASTERMIND study. Diabetic Medicine. 2015;32(S1):1-3.
13. Viberti G, Kahn SE, Greene DA, Herman WH, Zinman B, Holman RR, et al. A Diabetes Outcome Progression Trial (ADOPT). An international multicenter study of the comparative efficacy of rosiglitazone, glyburide, and metformin in recently diagnosed type 2 diabetes. 2002;25(10):1737-43.
14. Drucker DJ, Nauck MA. The incretin system: glucagon-like peptide-1 receptor agonists and dipeptidyl peptidase-4 inhibitors in type 2 diabetes. The Lancet. 2006;368(9548):1696-705.
15. Tahrani AA, Barnett AH, Bailey CJ. SGLT inhibitors in management of diabetes. The Lancet Diabetes & Endocrinology. 2013;1(2):140-51.
16. Chan A-W, Tetzlaff JM, Altman DG, Laupacis A, Gøtzsche PC, Krleža-Jerić K, et al. SPIRIT 2013 statement: defining standard protocol items for clinical trials. Annals of internal medicine. 2013;158(3):200-7.

For peer review only

For peer review only - http://bmjopen.bmj.com/site/about/guidelines.xhtml

17. Merck Sharp D. Summary of Product Characteristics: Januvia (sitagliptin phosphate monohydrate) available at https://www.medicines.org.uk/emc/product/7887/smpc. Electronic Medicines Compendium (EMC) 2020.
18. Napp Pharmaceuticals L. Summary of Product Characterstics: Invokana (Canagliflozin Hemihydrate) available at https://www.medicines.org.uk/emc/product/8855/smpc. Electronic Medicines Compendium (EMC). 2020.
19. Takeda. Summary of Product Characteristics: Actos Tablets available at https://www.medicines.org.uk/emc/product/1287/smpc. Electronic Medicines Compendium (EMC) 2019.
20. WHO. Use of glycated haemoglobin (HbA1c) in the diagnosis of diabetes mellitus. 2011. www.who.int/diabetes/publications/report-hba1c_2011.pdf.
21. Lund SS, Tarnow L, Stehouwer CD, Schalkwijk CG, Frandsen M, Smidt UM, et al. Targeting hyperglycaemia with either metformin or repaglinide in non-obese patients with type 2 diabetes: results from a randomized crossover trial. Diabetes, obesity & metabolism. 2007;9(3):394-407.
22. Dwan K, Li T, Altman DG, Elbourne D. CONSORT 2010 statement: extension to randomised crossover trials. BMJ. 2019;366:l4378.

For peer review only

For peer review only - http://bmjopen.bmj.com/site/about/guidelines.xhtml

TriMaster CONSORT Flow Diagram

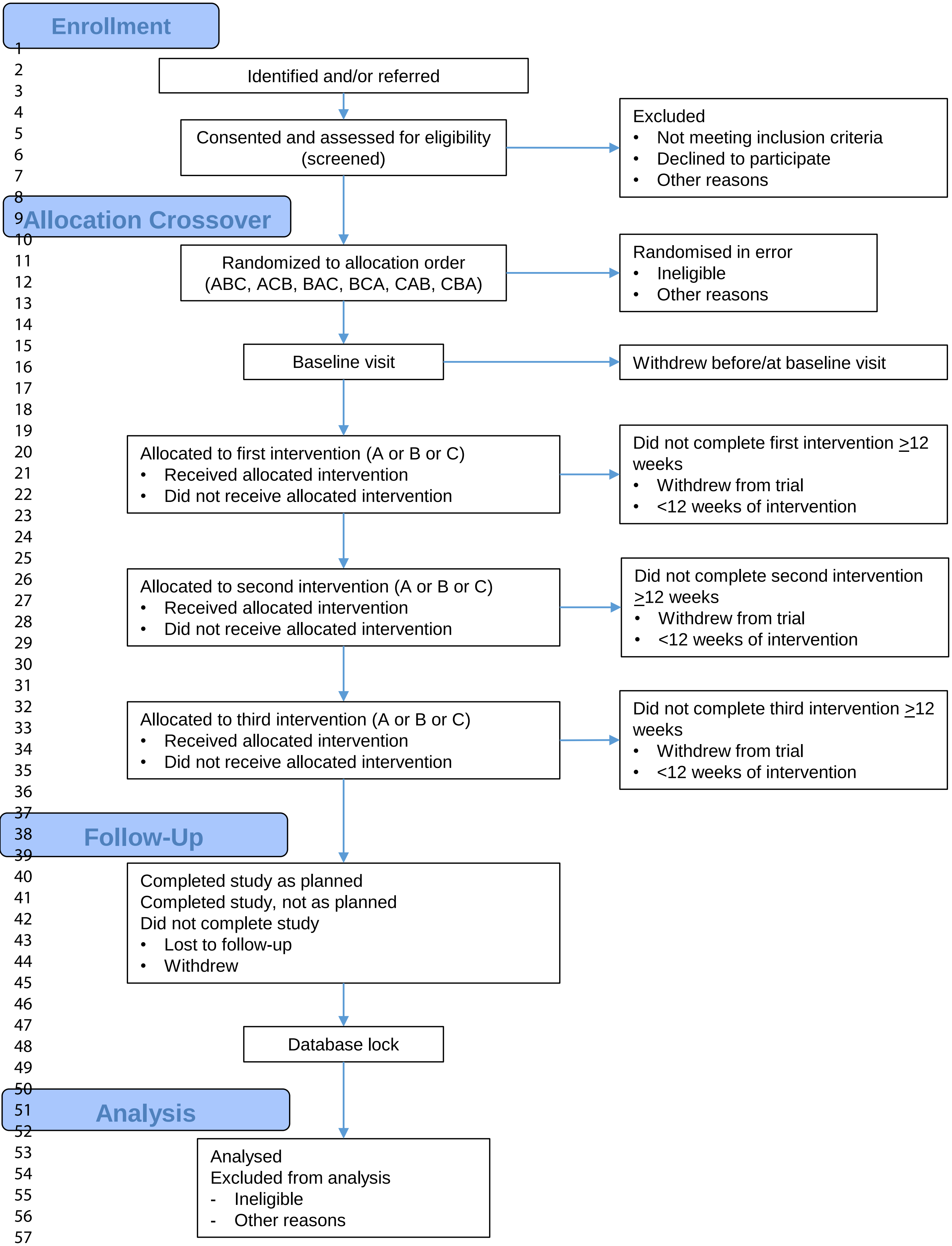

Figure 2. TriMaster Schedule of Assessment

	STUDY PERIOD							
	Pre-Screening	Screening	Baseline	Treatment			Follow up	
**TIMEPOINT****	$-t_1$	t_0	t_1 v1	t_2 v2	t_3 v3	t_4 v4	t_5 v5	t_6
**ENROLMENT:**								
Informed consent		X						
Medical History		X						
Concomitant medication		X						
Foot pulses		X						
Weight		X						
Height		X						
Eligibility Bloods		X	X[1]					
Urine Dip		X	X[1]					
Pregnancy Test (WOCBP)		X						
Randomisation			X					
**INTERVENTIONS:**								
Medication dispensing			X	X	X	X		
Medication return				X	X	X		
IMP accountability				X	X	X		
First IMP A/B/C				↔				
Second IMP A/B/C					↔			
Third IMP A/B/C						↔		
IMP code break (GP/patient)								X
**ASSESSMENTS:**								
Bloods (see assessment table)			X	X	X	X		
Urine Dip			X	X	X	X		
Genital swabs[2]			X	X	X	X		
Urine collection			X	X	X	X		
Pregnancy Test (WOCBP)			X	X	X	X		
DTSQ			X	X	X	X		
Patient experience			X	X	X	X	X	
Family history of diabetes			X					
Concomitant medication			X	X	X	X		
Weight			X	X	X	X		
Waist & hip measurement			X	X	X	X		
Body fat %			X	X	X	X		
Blood pressure			X	X	X	X		
Oedema of leg/ankle			X	X	X	X		
Adherence			X	X	X	X		
Side Effects			X	X	X	X		
Adverse events review			X	X	X	X		

[1] Where baseline visit takes place more than 2 weeks after screening visit, eligibility blood samples must be repeated.

[2] Optional procedure for participants at sites which have previously agreed to sample collection

For peer review only

Assessment	Screening	Visit 1 Baseline	Visit 1 MMTT	Visits 2-4
HbA1c	x	x		x
Full blood count	x			x
Creatinine	x	x		
ALT	x			
Glycosylated albumin		x	x	x
Glucose		x	x	x
C-peptide		x	x	x
Insulin		x	x	x
AST		x		x
Albumin				
Bilirubin				
GGT				
Triglycerides		x		x
Total, HDL and LDL cholesterol				
Autoantibodies – GAD, IA2, ZnT8		x		
Medication levels				x
B-type Natriuretic Peptide (BNP)		x		X

For peer review only

Reporting checklist for protocol of a clinical trial – TriMaster

Based on the SPIRIT guidelines: Chan A-W, Tetzlaff JM, Altman DG, Laupacis A, Gøtzsche PC, Krleža-Jerić K, Hróbjartsson A, Mann H, Dickersin K, Berlin J, Doré C, Parulekar W, Summerskill W, Groves T, Schulz K, Sox H, Rockhold FW, Rennie D, Moher D. SPIRIT 2013 Statement: Defining standard protocol items for clinical trials. Ann Intern Med. 2013;158(3):200-207

		Reporting Item	Page Number
**Administrative information**			
Title	#1	Descriptive title identifying the study design, population, interventions, and, if applicable, trial acronym	1
Trial registration	#2a	Trial identifier and registry name. If not yet registered, name of intended registry	2
Trial registration: data set	#2b	All items from the World Health Organization Trial Registration Data Set	Included in trial protocol and registry entry
Protocol version	#3	Date and version identifier	16
Funding	#4	Sources and types of financial, material, and other support	15
Roles and responsibilities: contributorship	#5a	Names, affiliations, and roles of protocol contributors	1 & 14
Roles and responsibilities: sponsor contact information	#5b	Name and contact information for the trial sponsor	Included in trial protocol and registry entry
Roles and responsibilities: sponsor and funder	#5c	Role of study sponsor and funders, if any, in study design; collection, management, analysis, and interpretation of data; writing of the report; and the	15

For peer review only

		decision to submit the report for publication, including whether they will have ultimate authority over any of these activities	
Roles and responsibilities: committees	#5d	Composition, roles, and responsibilities of the coordinating centre, steering committee, endpoint adjudication committee, data management team, and other individuals or groups overseeing the trial, if applicable (see Item 21a for data monitoring committee)	Included in trial protocol
**Introduction**			
Background and rationale	#6a	Description of research question and justification for undertaking the trial, including summary of relevant studies (published and unpublished) examining benefits and harms for each intervention	2
Background and rationale: choice of comparators	#6b	Explanation for choice of comparators	3
Objectives	#7	Specific objectives or hypotheses	4
Trial design	#8	Description of trial design including type of trial (eg, parallel group, crossover, factorial, single group), allocation ratio, and framework (eg, superiority, equivalence, non-inferiority, exploratory)	5
**Methods: Participants, interventions, and outcomes**			
Study setting	#9	Description of study settings (eg, community clinic, academic hospital) and list of countries where data will be collected. Reference to where list of study sites can be obtained	5
Eligibility criteria	#10	Inclusion and exclusion criteria for participants. If applicable, eligibility criteria for study centres and individuals who will perform the interventions (eg, surgeons, psychotherapists)	6

For peer review only

Interventions: description	#11a	Interventions for each group with sufficient detail to allow replication, including how and when they will be administered	9
Interventions: modifications	#11b	Criteria for discontinuing or modifying allocated interventions for a given trial participant (eg, drug dose change in response to harms, participant request, or improving / worsening disease)	9
Interventions: adherance	#11c	Strategies to improve adherence to intervention protocols, and any procedures for monitoring adherence (eg, drug tablet return; laboratory tests)	10
Interventions: concomitant care	#11d	Relevant concomitant care and interventions that are permitted or prohibited during the trial	7
Outcomes	#12	Primary, secondary, and other outcomes, including the specific measurement variable (eg, systolic blood pressure), analysis metric (eg, change from baseline, final value, time to event), method of aggregation (eg, median, proportion), and time point for each outcome. Explanation of the clinical relevance of chosen efficacy and harm outcomes is strongly recommended	8
Participant timeline	#13	Time schedule of enrolment, interventions (including any run-ins and washouts), assessments, and visits for participants. A schematic diagram is highly recommended (see Figure)	Figures 1 & 2
Sample size	#14	Estimated number of participants needed to achieve study objectives and how it was determined, including clinical and statistical assumptions supporting any sample size calculations	8
Recruitment	#15	Strategies for achieving adequate participant enrolment to reach target sample size	11

Methods: Assignment of interventions (for controlled trials)

Allocation: sequence generation	#16a	Method of generating the allocation sequence (eg, computer-generated random numbers), and list of any factors for stratification. To reduce predictability of a	9

For peer review only

		random sequence, details of any planned restriction (eg, blocking) should be provided in a separate document that is unavailable to those who enrol participants or assign interventions	
Allocation concealment mechanism	#16b	Mechanism of implementing the allocation sequence (eg, central telephone; sequentially numbered, opaque, sealed envelopes), describing any steps to conceal the sequence until interventions are assigned	10
Allocation: implementation	#16c	Who will generate the allocation sequence, who will enrol participants, and who will assign participants to interventions	10
Blinding (masking)	#17a	Who will be blinded after assignment to interventions (eg, trial participants, care providers, outcome assessors, data analysts), and how	10
Blinding (masking): emergency unblinding	#17b	If blinded, circumstances under which unblinding is permissible, and procedure for revealing a participant's allocated intervention during the trial	10

Methods: Data collection, management, and analysis

Data collection plan	#18a	Plans for assessment and collection of outcome, baseline, and other trial data, including any related processes to promote data quality (eg, duplicate measurements, training of assessors) and a description of study instruments (eg, questionnaires, laboratory tests) along with their reliability and validity, if known. Reference to where data collection forms can be found, if not in the protocol	Figure 2 & pages 7 & 11
Data collection plan: retention	#18b	Plans to promote participant retention and complete follow-up, including list of any outcome data to be collected for participants who discontinue or deviate from intervention protocols	10
Data management	#19	Plans for data entry, coding, security, and storage, including any related processes to promote data quality (eg, double data entry; range checks for data values). Reference to where details of data	12

For peer review only

		management procedures can be found, if not in the protocol	
Statistics: outcomes	#20a	Statistical methods for analysing primary and secondary outcomes. Reference to where other details of the statistical analysis plan can be found, if not in the protocol	13
Statistics: additional analyses	#20b	Methods for any additional analyses (eg, subgroup and adjusted analyses)	14
Statistics: analysis population and missing data	#20c	Definition of analysis population relating to protocol non-adherence (eg, as randomised analysis), and any statistical methods to handle missing data (eg, multiple imputation)	14

Methods: Monitoring

Data monitoring: formal committee	#21a	Composition of data monitoring committee (DMC); summary of its role and reporting structure; statement of whether it is independent from the sponsor and competing interests; and reference to where further details about its charter can be found, if not in the protocol. Alternatively, an explanation of why a DMC is not needed	14
Data monitoring: interim analysis	#21b	Description of any interim analyses and stopping guidelines, including who will have access to these interim results and make the final decision to terminate the trial	NA
Harms	#22	Plans for collecting, assessing, reporting, and managing solicited and spontaneously reported adverse events and other unintended effects of trial interventions or trial conduct	14
Auditing	#23	Frequency and procedures for auditing trial conduct, if any, and whether the process will be independent from investigators and the sponsor	14

For peer review only

Ethics and dissemination

Research ethics approval	#24	Plans for seeking research ethics committee / institutional review board (REC / IRB) approval	2
Protocol amendments	#25	Plans for communicating important protocol modifications (eg, changes to eligibility criteria, outcomes, analyses) to relevant parties (eg, investigators, REC / IRBs, trial participants, trial registries, journals, regulators)	16
Consent or assent	#26a	Who will obtain informed consent or assent from potential trial participants or authorised surrogates, and how (see Item 32)	11
Consent or assent: ancillary studies	#26b	Additional consent provisions for collection and use of participant data and biological specimens in ancillary studies, if applicable	NA
Confidentiality	#27	How personal information about potential and enrolled participants will be collected, shared, and maintained in order to protect confidentiality before, during, and after the trial	12
Declaration of interests	#28	Financial and other competing interests for principal investigators for the overall trial and each study site	16
Data access	#29	Statement of who will have access to the final trial dataset, and disclosure of contractual agreements that limit such access for investigators	14
Ancillary and post trial care	#30	Provisions, if any, for ancillary and post-trial care, and for compensation to those who suffer harm from trial participation	NA
Dissemination policy: trial results	#31a	Plans for investigators and sponsor to communicate trial results to participants, healthcare professionals, the public, and other relevant groups (eg, via publication, reporting in results databases, or other data sharing arrangements), including any publication restrictions	15
Dissemination policy: authorship	#31b	Authorship eligibility guidelines and any intended use of professional writers	NA

For peer review only

Dissemination policy: reproducible research	#31c	Plans, if any, for granting public access to the full protocol, participant-level dataset, and statistical code	15

Appendices

Informed consent materials	#32	Model consent form and other related documentation given to participants and authorised surrogates	Available on study website
Biological specimens	#33	Plans for collection, laboratory evaluation, and storage of biological specimens for genetic or molecular analysis in the current trial and for future use in ancillary studies, if applicable	Figure 2 & page 7

The SPIRIT checklist is distributed under the terms of the Creative Commons Attribution License CC-BY-ND 3.0. This checklist can be completed online using https://www.goodreports.org/, a tool made by the EQUATOR Network in collaboration with Penelope.ai

For peer review only

BMJ Open

TriMaster: Randomised Double-Blind Crossover study of a DPP4 inhibitor, SGLT2 inhibitor and thiazolidinedione as second or third line therapy in patients with Type 2 diabetes who have suboptimal glycaemic control on metformin treatment with or without a sulfonylurea – a MASTERMIND study protocol

Journal:	*BMJ Open*
Manuscript ID	bmjopen-2020-042784.R1
Article Type:	Protocol
Date Submitted by the Author:	24-Nov-2020
Complete List of Authors:	Angwin, Catherine; University of Exeter, Medical School<br>Jenkinson, C; University of Exeter,<br>Jones, Angus; University of Exeter, The Institute of Biomedical & Clinical Science<br>Jennison, Christopher; University of Bath<br>Henley, W; University of Exeter<br>Farmer, Andrew; University of Oxford, Nuffield Department of Primary Care Health Sciencesd<br>Sattar, Naveed; University of Glasgow, Institute of Cardiovascular and Medical Sciences<br>Holman, Rury; University of Oxford Medical Sciences Division, Radcliffe Department of Medicine<br>Pearson, Ewan; University of Dundee<br>Shields, Beverley; University of Exeter<br>Hattersley, Andrew; University of Exeter
**Primary Subject Heading**:	Diabetes and endocrinology
Secondary Subject Heading:	Research methods
Keywords:	DIABETES & ENDOCRINOLOGY, Clinical trials < THERAPEUTICS, THERAPEUTICS

SCHOLARONE™
Manuscripts

I, the Submitting Author has the right to grant and does grant on behalf of all authors of the Work (as defined in the below author licence), an exclusive licence and/or a non-exclusive licence for contributions from authors who are: i) UK Crown employees; ii) where BMJ has agreed a CC-BY licence shall apply, and/or iii) in accordance with the terms applicable for US Federal Government officers or employees acting as part of their official duties; on a worldwide, perpetual, irrevocable, royalty-free basis to BMJ Publishing Group Ltd ("BMJ") its licensees and where the relevant Journal is co-owned by BMJ to the co-owners of the Journal, to publish the Work in this journal and any other BMJ products and to exploit all rights, as set out in our licence.

The Submitting Author accepts and understands that any supply made under these terms is made by BMJ to the Submitting Author unless you are acting as an employee on behalf of your employer or a postgraduate student of an affiliated institution which is paying any applicable article publishing charge ("APC") for Open Access articles. Where the Submitting Author wishes to make the Work available on an Open Access basis (and intends to pay the relevant APC), the terms of reuse of such Open Access shall be governed by a Creative Commons licence – details of these licences and which Creative Commons licence will apply to this Work are set out in our licence referred to above.

Other than as permitted in any relevant BMJ Author's Self Archiving Policies, I confirm this Work has not been accepted for publication elsewhere, is not being considered for publication elsewhere and does not duplicate material already published. I confirm all authors consent to publication of this Work and authorise the granting of this licence.

For peer review only

TriMaster: Randomised Double-Blind Crossover study of a DPP4 inhibitor, SGLT2 inhibitor and thiazolidinedione as second or third line therapy in patients with Type 2 diabetes who have suboptimal glycaemic control on metformin treatment with or without a sulfonylurea – a MASTERMIND study protocol

Authors: CD Angwin[1,2], CE Jenkinson[1,2], AG Jones[1,3], C Jennison[4], WE Henley[5], AJ Farmer[6], N Sattar[8], RR Holman[5], ER Pearson[4], BM Shields[1,2]*, AT Hattersley[1,3]* for the MASTERMIND consortium

these authors contributed equally

Affiliations:

[1]Institute of Biomedical & Clinical Sciences, University of Exeter Medical School, Exeter, UK

[2]NIHR Exeter Clinical Research Facility, Royal Devon and Exeter NHS Foundation Trust, Exeter, UK

[3]Diabetes and Endocrinology, Royal Devon and Exeter Hospital NHS Foundation Trust, Exeter, UK

[4]Department of Mathematical Sciences, University of Bath, Bath, UK [4]Division of Population Health and Genomics, Ninewells Hospital and School of Medicine, University of Dundee, Dundee, Scotland, UK

[5]Institute of Health Research, University of Exeter Medical School, Exeter, UK

[6]Nuffield Department of Primary Care Health Sciences, University of Oxford, Oxford, UK

[7]Institute of Cardiovascular and Medical Sciences, University of Glasgow, Glasgow, UK

[8]Radcliffe Department of Medicine Diabetes Trial Unit (OCDEM), University of Oxford, Oxford, Oxfordshire, UK

Corresponding authors:

Prof Andrew Hattersley A.T.Hattersley@exeter.ac.uk

Dr Beverley Shields B.Shields@exeter.ac.uk

Word count: 4019 – excluding abstract, references, figures and tables

For peer review only

For peer review only - http://bmjopen.bmj.com/site/about/guidelines.xhtml

ABSTRACT

Introduction

Pharmaceutical treatment options for patients with type 2 diabetes (T2DM) have increased to include multiple classes of oral glucose-lowering agents but without accompanying guidance on which of these may most benefit individual patients. Clinicians lack information for treatment intensification after first-line metformin therapy. Stratifying patients by simple clinical characteristics may improve care by targeting treatment options to those in whom they are most effective. This academically-designed and run three-way crossover trial aims to test a stratification approach using three standard oral glucose-lowering agents.

Methods and analysis

TriMaster is a randomised, double-blind, crossover trial taking place at up to 25 clinical sites across England, Scotland and Wales. 520 patients with T2DM treated with either metformin alone, or metformin and a sulfonylurea who have HbA_{1c} >58mmol/mol will be randomised to receive 16 weeks each of a DPP4 inhibitor, SGLT2 inhibitor and thiazolidinedione in random order. Participants will be assessed at the end of each treatment period, providing clinical and biochemical data, and their experience of side effects. Participant preference will be assessed on completion of all 3 treatments. The primary endpoint is HbA_{1c} after 4 months of therapy (allowing a range of 12-18 weeks for analysis). Secondary endpoints include participant-reported preference between the three treatments, tolerability and prevalence of side effects.

Ethical approval: This study was approved by NHS Health Research Authority (HRA) Research Ethics Committee South Central – Oxford A, study 16/SC/0147. Written informed consent will be obtained from all participants. Results will be submitted to a peer-reviewed journal and presented at relevant scientific meetings. A lay summary of results will be made available to all participants.

Trial registration number: ISRCTN: 12039221. EudraCT: 2015-002790-38. ClinicalTrials.gov: NCT02653209.

For peer review only - http://bmjopen.bmj.com/site/about/guidelines.xhtml

ARTICLE SUMMARY

Strengths and limitations of this study:

- This is the first blinded three-way crossover trial of glucose-lowering therapies in T2DM, allowing comparison of short term treatment response and side effects across 3 agents within the same individuals
- This study design enables assessment of stratification allowing for within-person variation in response
- This will be the first study to assess patient preference for choosing between 3 glucose-lowering therapies
- A limitation is that only short-term glycaemic response and side effects can be assessed in a study of this design

BACKGROUND AND RATIONALE

In recent years the choice of therapies designed to lower glucose in patients with T2DM has increased(1) but there remains limited information as to which patients may respond well, moderately, or poorly to any of the treatment options(2, 3). Treatment intensification is recommended in a step-wise approach, with guidelines usually including a number of different agents after metformin in those without established cardiac or renal disease(2, 4).

T2DM is a heterogenous condition and response to glucose-lowering therapy appears to vary substantially between individuals. Therefore identification of subgroups of patients who respond well or poorly to a specific therapy, or with an altered risk of treatment-specific side effects, could improve targetting of treatment. This stratified approach to therapy is most likely to be successful if based on clinical characteristics and biomarkers that are readiliy available in routine clinical care: T2DM is common, most therapies are relatively inexpensive and most management is undertaken in primary care, therefore stratification based on expensive biomakers, or those with limited availability, is unlikely to be widely adopted.

A number of previous studies have shown simple clinical characteristics and biomarkers are associated with variation in glycaemic response for individual therapies(5-8). However, to be most useful for stratification a marker needs to predict differential response between therapies(9). Work by the MASTERMIND consortium using routine and trial data has strengthened the evidence that clinical features are associated with differential glycaemic response to DPP4-inhibitors, SGLT2-inhibitors and thiazolidinediones (10-12). Analysis of data from the UK Clinical Practice Research

For peer review only - http://bmjopen.bmj.com/site/about/guidelines.xhtml

For peer review only

Datalink (CPRD) and ADOPT trial showed that sex and BMI above and below 30 were associated with differential glycaemia response between sulfonylureas and thiazolidinediones (10). In addition, individuals within a normal estimated glomerular filtration rate (eGFR) range, with a higher eGFR show a better glycaemia response to Sodium-glucose co-transporter-2 (SGLT2) inhibitors whilst individuals with a lower eGFR may have a better glycaemic response when taking a Dipeptidyl peptidase-4 (DPP4) inhibitor (Janssen, personal communication from MASTERMIND industry group, 2014). The features identified (sex, obesity and renal function), are routinely measured at low cost, meaning potential stratification using these characteristics could be easily implemented in clinical practice.

TriMaster aims to test potential glycaemic therapy stratification in T2DM using response to three standard glucose-lowering agents. It will determine whether subgroups defined by routinely measured features respond to a greater or lesser degree (with regard to glycaemic change) to DPP4-inhibitors, SGLT2-inhibitors and thiazolidinediones, and provide a resource for further investigation of stratification between these therapies in the future.

These therapies were selected on the basis of differential response seen in pilot studies, and the choice of available oral third-line therapies at the start of the study.

Hypotheses:

The trial is designed with the following hypotheses:

1. Patients with insulin resistance, characterised clinically by a raised BMI (>30kg/m^2), compared to non-obese patients will: (a) respond well to pioglitazone, a thiazolidinedione that works as an insulin sensitiser(13); (b) respond less well to sitagliptin, a DPP4-inhibitor which works through stimulating endogenous insulin secretion post-prandially(14).

2. Patients with modestly reduced eGFR (60-90 mls/min/1.73m^2), compared to those with eGFR >90 mls/min/1.73m^{2,} will: (a) respond less well to canagliflozin, an SGLT2 inhibitor, which works through inhibiting the active reabsorption of glucose in the proximal tube(15), as the reduced eGFR will reduce the glucose-lowering efficacy; (b) respond well to sitagliptin, a DPP4 inhibitor that is renally cleared, as the reduced eGFR will increase plasma DPP4 inhibitor concentrations.

PRIMARY OBJECTIVES

To test two hypotheses of drug response stratification based on drug mechanism of action and pharmacokinetics to answer the following clinical questions:

For peer review only - http://bmjopen.bmj.com/site/about/guidelines.xhtml

1. Do obese patients (BMI >30kg/m^2), compared to non-obese patients, achieve a lower HbA_{1c} when assigned to pioglitazone rather than sitagliptin?
2. Do patients with an eGFR 60-90 mls/min/1.73m^2 achieve a lower HbA_{1c}, compared to patients with an eGFR >90 mls/min/1.73m^2, when assigned to sitagliptin rather than canagliflozin?

SECONDARY OBJECTIVES

The design of the study provides people with T2DM the unusual opportunity to try a panel of three available glucose-lowering therapies and to express a preference based on their experience of each. The study's secondary objectives are to determine:

1. Patient treatment preference within hypothesised strata and overall.
2. Prevalence of side effects within hypothesised strata and for specific drugs, to include: weight gain, hypoglycaemia, oedema, genital tract infection and discontinuation of therapy.
3. Predefined test of sex heterogeneity with pilot data suggesting females are more likely to show an improved response relative to males for pioglitazone.
4. Tolerability of treatments within hypothesised strata and overall.

METHODS AND ANALYSIS

We have used the SPIRIT reporting guidelines in the design of the protocol and preparation of this paper(16) (Appendix 1).

Overview of trial design

TriMaster is a phase four, academically-designed and run, multi-centre, randomised, double-blind, 12-month crossover trial of a DPP4 inhibitor (sitagliptin), thiazolidinedione (pioglitazone) and SGLT2 inhibitor (canagliflozin) as a second or third line therapy in patients with T2DM who have suboptimal glycaemic control on metformin alone or metformin and sulfonylurea (Figure 1 – TriMaster Consort diagram). The three-way crossover will be undertaken as an efficient, faster and more cost-effective approach to address both hypotheses, requiring fewer participants than performing two 2-way cross over studies.

520 participants with T2DM will be recruited, aged 30-80 years on stable doses of metformin alone or metformin and a sulfonylurea with glycated haemoglobin (HbA_{1c}) >58mmol/mol (>7.5%) and ≤110mmol/mol (≤12.2%). Each participant will attend one screening and, if eligible, five research

For peer review only - http://bmjopen.bmj.com/site/about/guidelines.xhtml

visits over a 12-month period (50-60 weeks max/min visit windows). They will receive the three blinded second and third-line oral therapies in random order for 16-18 weeks each, with no washout period between therapies. Participant feedback from pilot studies found repeated washout periods increased rates of withdrawal due to poorly tolerated hyperglycaemia. Once stopped, none of the three glucose-lowering agents used in this study have a continuing glucose-lowering effect beyond four weeks (all three drugs have half-lives between 7 and 14 hours so their effects should be negligible after a week)(17-19) and HbA_{1c} measurement reflects glycaemia over the preceding eight to twelve-week period. HbA_{1c} measurements taken after 16 weeks will therefore allow a 4-week 'wash-in' period and effectively reflect response to each treatment period.

Participants will each act as their own control, and on completion of all three treatments will be asked to rank the treatments taken in order of preference. Eligible participants will be recruited at 20-25 UK sites; the trial is sponsored by the Royal Devon and Exeter NHS Foundation Trust and hosted at the National Institute for Health Research (NIHR) Exeter Clinical Research Facility. A full list of recruiting sites will be available via the ISRCTN registration.

Eligibility criteria

All potential participants will undergo a formal screening visit to assess and confirm eligibility as listed in Table 1.

Outcome measures

In line with WHO guidelines, response to therapy will be assessed by measurement of glycated haemoglobin (HbA_{1c})(20). The primary outcome is the HbA_{1c} value achieved after each 16-week treatment period. Should a participant be unable to complete a full 16-week treatment period, HbA_{1c} will be measured and included in the main analysis if the participant has taken the study drug for at least 12 weeks.

Secondary outcomes will be participant-reported preference between the three treatments, tolerability of the three treatments and prevalence of side effects. In addition, we will explore sex differences in response to the three drugs.

Participant willingness to continue a drug long term will be recorded at the end of each treatment arm. Treatment preference, taken as a ranking of the three study drugs will be recorded at study completion. To inform this decision, in addition to their experience on each drug, clinical information

For peer review only - http://bmjopen.bmj.com/site/about/guidelines.xhtml

including HbA_{1c} measurements and weight change will be fed-back to each participant. Frequency and severity of side effects will be recorded throughout the study alongside the Diabetes Treatment Satisfaction Questionnaire (DTSQ) to allow a formal validated assessment of participant satisfaction.

HbA_{1c} assessment during the study will be performed in local NHS laboratories to ensure results are available for screening, and to inform final patient preference. Central laboratory analysis will be undertaken at the Exeter Clinical Laboratory at the Royal Devon & Exeter NHS Foundation Trust for all other non-safety sample analysis. All analyses are routine biochemistry tests available in the NHS test repertoire. All assays are CE marked, fully validated and accredited by the United Kingdom Accreditation Service (UKAS).

Sample size

The primary outcome is HbA_{1c} at the end of each treatment period.

This trial aims to test whether participants in a particular strata (S) respond differently to drug A and drug B compared with patients not in the strata (N). The primary outcome is the HbA_{1c} measurement after 4 months of each drug (Table 2).

The null hypothesis is that the difference in achieved HbA_{1c} for the two drugs will be similar for the two groups of participants (i.e. $HbA1c_{SA}$-$HbA1c_{SB}$ = $HbA1c_{NA}$-$HbA1c_{NB}$ in Table 2).

In a crossover trial of metformin versus repaglinide the standard deviation (SD) of change in HbA_{1c} on the two different therapies was 8.7mmol/mol(21). Analysis of Clinical Practice Research Datalink (CPRD) showed obese patients respond better to TZDs and non-obese patients respond better to DPP4is, with an overall difference in response between strata of 3.1mmol/mol (equivalent to 0.36SDs). Similarly, higher eGFR >90ml/min/1.73m² is associated with a better HbA_{1c} response to SGLT2i, whilst patients with an eGFR 60-90ml/min/1.73m² had a lower response to the SGLT2i and higher response to DPP4i with an overall difference in response between strata of 3.0mmol/mol (equivalent to 0.35SDs) (Janssen, personal communication, 2014).

Using 90% power, alpha=0.05, to detect a difference of 0.35SDs we require 172 participants in each stratum, 344 in total. To allow for the possibility of unequal numbers in each stratum, the sample size has been increased to 358, assuming a 60:40 split (T2DM population CPRD 52:48 for both strata; a conservative withdrawal rate of 15% increases the study sample size to 422. To allow for

For peer review only - http://bmjopen.bmj.com/site/about/guidelines.xhtml

participants excluded from primary analysis due to fewer than 12 weeks on one or more study drugs (estimated at 19%), we will increase the total sample size for the study to 520.

Investigational Medicinal Product (IMP)

Trial interventions were chosen in line with UK NICE guidelines for first and second intensification of drug treatment in patients with T2DM(2). The three drugs will be provided to participants in a blinded format and randomised order at the starting dose indicated in the British National Formulary (BNF); sitagliptin 100mg, canagliflozin 100mg, pioglitazone 30mg.

IMPs will be supplied directly to recruiting site pharmacies by Tayside Pharmaceuticals, Dundee, UK. Tablets will be over-encapsulated in a hard gelatin capsule so that the IMPs are near identical in size and colour, and packed into bottles and distributed to each recruitment site (see randomisation section for further details).

Participants will be instructed to take one capsule, once daily, alongside their existing diabetes treatment and usual medications. They will be given a Drug Information Sheet in place of a standard Summary of Product Characteristics stating the expected side effects of all three treatments. To allow feasible visit windows and prevent participants running out of IMP, each IMP bottle will contain 126 capsules, the equivalent of 18 weeks' medication.

Where a participant is unable to tolerate a therapy, they will move to the next IMP in their randomised order, providing they remain clinically safe to continue in the study. Dose modification, reduction or delay will not be permitted due to the blinded nature of the trial. Participants will be asked to return the IMP bottle and all unused capsules; research staff will perform a capsule count for adherence and accountability purposes.

Randomisation, allocation and blinding

The study has six treatment sequence permutations: ABC, ACB, BAC, BCA, CAB and CBA; participants will be randomly allocated to one of the six sequences when confirmed as eligible in the study database. The study is double-blind and all clinical, participant and laboratory assessments will be made prior to database lock, final analysis, and unblinding of the drug order.

A block randomisation list (block size 12) will be created using Statsdirect by the Trial Statistician and the randomisation seed recorded. The randomisation list will be provided to the study database

For peer review only - http://bmjopen.bmj.com/site/about/guidelines.xhtml

team who will randomly allocate blocks of 12 to each of the recruitment sites (to ensure balance between the 6 treatment orders at each site), with the allocation remaining blinded to the rest of the study team. The IMP supplier will provide each site with blocks of 12 drugs (4 of each of the 3 study drugs) with the 12 bottle IDs labelled in random order to avoid the drug type being easily identifiable. The bottle IDs and contents assigned to each recruitment site will be recorded in the study database and accessible only to the database team.

To ensure allocation concealment, randomisation will be centralised via the study database. Eligibility will be confirmed by research teams and the participant randomised to a blinded treatment order, allocated by the study database. Prior to research visits one, two and three, the database will allocate the next available IMP bottle of the correct drug type held at that site, according to this treatment order. Study prescriptions detailing the allocated bottle ID will be processed and dispensed by the site clinical trials pharmacy.

Participants will not be recruited against specific strata; BMI and eGFR defined stratum will be monitored as recruitment and randomisation progresses. Data on the distribution will be provided to the Data Monitoring Committee and if enrolment is unevenly distributed to an extent that the study hypotheses cannot be robustly tested, the relevant stratum may be 'switched off' by the Data Programmer to prevent further randomisation into the relevant strata.

Participants who withdraw before randomisation will be replaced. However, once randomised, their data will be included in analysis. To maintain data quality and trial integrity, unblinding via code breaks will occur only in exceptional circumstances where knowledge of the IMP is deemed essential for the correct clinical management of the participant, a medical emergency where someone other than the participant has taken the IMP, or where this information is needed to establish expectedness of a potential Suspected Unexpected Serious Adverse Reaction (SUSAR). The emergency code break table is available electronically on the study database requiring multiple confirmation steps to avoid accidental unblinding, and on paper in the central coordinating centre. In the event a code break is required this will be done by a member of staff independent of the main trial team. A study involvement card with study ID, IMP details, and contact information for local and central emergency unblinding will be provided to all participants.

Study visits and procedures

Figure 2 illustrates the schedule of assessments.

For peer review only - http://bmjopen.bmj.com/site/about/guidelines.xhtml

Identification and recruitment

Potential participants will be identified through primary and secondary care, research databases and direct clinician referral, provided with an information sheet, and invited to attend a screening visit. Following informed consent (Appendix 2) by trained and delegated research staff, clinical characteristics (height, weight, waist and hip circumference), medical history, concomitant medication details and non-fasting blood samples will be collected to confirm eligibility. Eligible participants will be randomised into the trial, assigned a unique Study ID and allocated a drug order.

Baseline visit

Within two weeks of screening, participants will attend a fasting baseline research visit. Baseline physiological data will be collected, along with self-reported compliance to existing diabetes medication. Participant's personal priorities in choosing between treatment options and experience of side effects on current treatment will also be recorded. Participants will have underlying pathophysiology assessed in a two-hour mixed-meal tolerance test (MMTT) using a standard meal drink (Fortisip). The meal test will be undertaken using 250ml of Nutricia Fortisip or 160ml of Nutricia Fortisip Compact. Equivalent products may be used where Fortisip cannot be tolerated. Blood and urine samples will be collected for analysis and future biomarker discovery at baseline, and then at 30-minute intervals (0, 30, 60, 90 and 120 minutes) following the meal drink. Participants at the central Exeter site will also be invited to provide a self-collected genital swab sample to identify development of sub-clinical colonisation of candida or bacteria.

Subsequent research visits will take place after 16-18 weeks of study treatment. However, patients will be offered the opportunity to stop a treatment early and move onto the next treatment period if they are unable to tolerate the therapy. Visits will repeat the baseline physiological measurements with samples collected at a single time point. Participants will provide fasting blood samples for immediate measurement of HbA_{1c}, and subsequent assessment to include fasting glucose, c-peptide, insulin, glycosylated albumin, creatinine, lipid profile and drug levels. Weight, blood pressure, adherence and data about patient experience will also be collected, including perceived side effects, preparedness to remain on the drug long term, and health-related quality of life. Where collected at baseline, subsequent genital swabs will be repeated at study visits two to four.

Case Report Forms (CRFs) will be completed at recruiting centres and securely transferred to the central team via nhs.net email. OpenText TeleForm will be used for data capture and transfer to the

For peer review only - http://bmjopen.bmj.com/site/about/guidelines.xhtml

study database. Identifiable data will be securely stored at recruiting centres, research data transferred to Exeter will be accessed only by delegated members of the research team.

Questionnaires - Participant preference

On completion of the third study drug, participants will be provided with a summary of their previous assessments of each therapy. At a final study visit, participants will first rank the study drugs based solely on their own experience on treatment. HbA_{1c} and weight data for each drug period will then be provided by the research team, and a repeat ranking recorded. This summary assessment was developed with the TriMaster Patient Involvement Group and the Peninsula Research Bank Lay Committee members. Endpoints of willingness to remain on study drug long term, and impact on daily life were identified as the best representations to capture participant preference for the study.

A final version of drug preference and clinical data will be provided to the participant and their clinician. This document, provided directly by the Exeter CTU team to ensure research teams remain blinded throughout, will contain details of the unblinded study drugs A, B and C to inform future treatment choices. All study procedures will occur within the 50-60 week trial period but permission will be requested to contact participants after primary analysis is complete to assess future treatment choice.

Participants will also complete the Diabetes Treatment Satisfaction Questionnaire (DTSQ) at baseline and the change version (DTSQc) after each treatment period to collect validated satisfaction scores.

Statistical analysis

Analyses and reporting will follow CONSORT guidance for randomised crossover trials(22). This study is not designed to test drug efficacy but the effectiveness of stratification. Therefore, only patients completing at least 12 weeks on therapy (sufficient to allow HbA_{1c} to reflect glycaemia control on the drug) will be included in primary analysis. In addition, we will perform a secondary analysis of tolerability examining whether the proportion of participants completing at least 12 weeks differs for each drug, both within strata and overall.

Prior to main analysis, we will determine whether there is any evidence of carryover or period effects. Any carryover effect identified will be reported but not adjusted for in subsequent analysis. Period effects will be reported and adjusted for. We do not anticipate treatment effect carryover

For peer review only - http://bmjopen.bmj.com/site/about/guidelines.xhtml

and have designed the study to limit potential carryover (as far as possible). Any period effect in the maximum 8 months between on treatment HbA_{1c}s is likely to be minimal as mean progression is 1.0 mmol/mol/year (personal communication E Pearson, data from GoDARTs population data).

There will be two primary analyses, one for each of the study hypotheses. For each hypothesis the primary analysis will be to assess whether the difference in achieved HbA_{1c} measurements for the two drugs is similar for the two groups of participants. The two hypotheses will be tested separately using linear mixed effects models to compare the strata on the two drugs of interest, with a random effects term for the participant. The key contrast of interest is the drug*strata interaction, where the strata is either obesity group or eGFR group. To determine whether there is a difference between drug classes in terms of the overall achieved HbA_{1c} after 4 months on each of the drugs, we will fit an additional model. Drug will be a factor and coded as a dummy variable as the comparison will be across 3 rather than 2 drug classes. Least square means will be extracted for the 3 drugs. Similar analysis will be carried out with weight after 4 months as the outcome.

In addition, we will examine the distribution of side effects reported across each of the 3 drugs. However, given the total numbers reporting each individual side effect will likely be small, we anticipate this will largely be descriptive, examining proportion of side effects observed with each drug.

For analysis of patient preference, we will only analyse the dataset where the participants have tried all 3 drugs. The mean rank for each drug will be calculated and tested against the null hypothesis that there is not a preferred drug and therefore the expected value of the rank for a given drug will be 2. Further investigation of patient preference will be exploratory.

Reasons for missing data will be documented and the baseline characteristics of those with and without missing data compared.

Monitoring

Due to the nature of standard diabetes treatments, it is expected that participants will experience some mild adverse events or reactions. These will be recorded at research visits and reported on a study-wide basis to the sponsor and Data Monitoring Committee at regular intervals.

For peer review only - http://bmjopen.bmj.com/site/about/guidelines.xhtml

Serious Adverse Reactions where the IMP is assessed as having possible, probable or definite causality will be unblinded to enable full evaluation of expectedness in the context of the relevant safety profile. Independent auditing of the trial will be arranged by the sponsor, in addition to sponsor review.

Oversight committees

The study will be run by a Trial Management Group (TMG) with oversight from an independent Trial Steering Committee (TSC) and Data Monitoring Committee (DMC). These committees comprise of independent experts in diabetes and statistical methodology and patient representatives and will meet regularly to monitor the scientific integrity and safety of the trial and provide independent advice. To ensure the safety of participants, the DMC will review unblinded safety data.

Ethics and Dissemination

This study has been reviewed and received ethics approval from the NHS Health Research Authority (HRA) Research Ethics Committee South Central – Oxford A, study 16/SC/0147. The Clinical Trial Application was reviewed and approved by the UK Medicines and Healthcare products Regulatory Agency (MHRA) under EudraCT reference: 2015-002790-38. All substantial and non-substantial amendments have received approval from HRA, REC and MHRA before implementation. The protocol has been registered with ClinicalTrials.gov (NCT02653209) and ISRCTN (12039221) (Trial registration dataset in Appendix 3).

All participants will be provided verbal and written information about the study prior to providing written informed consent and will be free to withdrawn at any time without affecting current or future clinical treatment.

Changes to protocol

The study was first registered with Clinicaltrials.gov (NCT02653209) on 12.01.2016 and ISRCTN (ISRCTN12039221) on 02.11.2016. Approved protocol amendments are in Table 3.

Dissemination

Data and results related to protocol-derived outcomes will be published in peer-reviewed journals by the Chief Investigator on behalf of the MASTERMIND consortium and presented at scientific meetings. Anonymous trial data will be shared within the MASTERMIND consortium and after publication of results, data will be securely deposited in Exeter's institutional repository and made available on request via the consortium's data access group. A lay summary will be provided to all study participants and made available on the study website, and public registries.

For peer review only - http://bmjopen.bmj.com/site/about/guidelines.xhtml

For peer review only

Patient and Public Involvement

Patients were involved in the design and conduct of this study. Following pilot studies, the TriMaster Patient Involvement Group provided feasibility feedback on the study design and the outcome measures used to record patient preference and experience of the study drugs. This group and the Peninsula Research Bank Lay Committee members assisted in the design of patient-facing documents, including consent forms, study and drug information sheets and data collection forms to assess patient preference.

Funding statement:

This work is part of the MASTERMIND (MRC APBI Stratification and Extreme Response Mechanism IN Diabetes) consortium and is supported by the UK Medical Research Council study grant number MR/N00633X/1. The funder and sponsor had no role in study design, and will not have a role in collection, management, analysis and interpretation of data, or decision to submit results for publication. The TriMaster trial is supported by the National Institute for Health Research (NIHR) Exeter Clinical Research Facility. The views expressed are those of the authors and not necessarily those of the MRC, NHS, the NIHR or the Department of Health. ERP holds a Wellcome Trust New Investigator Award (102820/Z/13/Z). AGJ is supported by an NIHR Clinician Scientist award (CS-2015-15-018). NS is supported by a BHF Centre of Excellence Award (RE/18/6/34217). RRH is an Emeritus National Institute for Health Research Senior Investigator. ATH is a Wellcome Senior Investigator (098395/Z/12/Z) and a Senior Investigator at the NIHR. ATH, AGJ, BS and CA are supported by the NIHR Exeter Clinical Research Facility. AF is an NIHR Senior Investigator and receives support from NIHR Oxford Biomedical Research Centre

Authors' contributions: state how each author was involved in writing the protocol:

ATH, BMS, AGJ, ERP, RRH, CJ, AJF, NS, WEH and CDA were involved in conception and trial design. CDA, CEJ, BMS and AGJ were involved in drafting of the article. ATH, BMS, AGJ, ERP, RRH, CEJ, AJF, CJ, WEH and NS were involved in critical revision of the article. All the authors were involved in final approval of the article.

Competing interest statement

ERP has received Honoraria from Lilly. NS has consulted for Amgen, Astrazeneca, Boehringer Ingelheim, Eli-Lilly, Napp, NovoNordisk, Sanofi and Pfizer and received grant funding from Boehringer Ingelheim. RRH reports research support from AstraZeneca, Bayer and Merck Sharp & Dohme, and personal fees from Bayer, Intarcia, Merck Sharp & Dohme, Novartis and Novo Nordisk

For peer review only - http://bmjopen.bmj.com/site/about/guidelines.xhtml

outside the submitted work. CJ has consulted for AstraZeneca, Boehringer Ingelheim, NovoNordisk and Sanofi. WEH has received grant funding from IQVIA and travel funds from Eisai.

For peer review only

For peer review only - http://bmjopen.bmj.com/site/about/guidelines.xhtml

Figures:

1. TriMaster Consort Diagram
2. TriMaster Schedule of Assessment

Supplementary Appendices

1. SPIRIT checklist
2. Patient consent form for the trial
3. Trial registration dataset

For peer review only

Table 1

Table 1. Eligibility Criteria

**Inclusion criteria**	**Exclusion criteria**
• Clinical diagnosis of T2DM<br>• Age ≥30 and ≤80 years<br>• Currently treated with one or two classes of oral glucose-lowering therapy (given either as separate or combined medications), that do not include a DPP4-inhibitor, a SGLT2-inhibitor or a thiazolidinedione<br>• Diabetes duration ≥12 months<br>• No change in diabetes treatment (new treatments or dose change) within previous 3 months<br>• HbA_{1c} > 58mmol/mol (>7.5%) and ≤110mmol/mol (≤12.2%)<br>• eGFR ≥ 60mls/min/1.73m² • Able and willing to give informed consent	• Changes in glucose-lowering therapy or dose within last 3 months<br>• ALT >2.5 x upper limit of the assay normal range (ULN) or known liver disease, specifically bilirubin >30 µmol/L that is associated with other evidence of liver failure<br>• Insulin treated within the last 12 months<br>• Treated with study dugs within the last 3 months<br>• Limb ischaemia shown by absence of both pulses in one or both feet<br>• Currently treated with corticosteroids<br>• Currently treated with rifampicin, gemfibrozil, phenytoin and carbamazepine<br>• Active infection (any infection requiring antibiotics)<br>• Foot ulcer requiring antibiotics within previous three months<br>• Recent (within 3 months) significant surgery or planned surgery (excluding minor procedures)<br>• Acute cardiovascular episode (angina, myocardial infarction, stroke, transient ischemic episode) occurring within the previous 3 months<br>• History of heart failure<br>• Current use of loop diuretic therapy (Furosemide or Bumetanide)<br>• History of bladder carcinoma

For peer review only - http://bmjopen.bmj.com/site/about/guidelines.xhtml

	• Current/ongoing investigation for macroscopic haematuria<br>• History of diabetic ketoacidosis<br>• History of pancreatitis<br>• Pregnant, breastfeeding or planning a pregnancy over the study period<br>• Concurrent participation on another Clinical Trial of an Investigational Medicinal Product (CTIMP) where the IMP is currently being taken, or without sufficient washout period (five times the longest half-life of the study IMPs) and without consultation with the CTIMP research team<br>• Unable or unwilling to give informed consent<br>• Females of childbearing potential must be willing to use an effective method of contraception from the time consent is signed until seven days after treatment discontinuation. A negative pregnancy test is required within seven days prior to treatment initiation and will be required for continuation at each study visit

For peer review only

For peer review only - http://bmjopen.bmj.com/site/about/guidelines.xhtml

Table 2: Differences in response between two drugs and two strata in the crossover trial. The null hypothesis is that the difference in achieved HbA_{1c} for the two drugs will be similar for the two groups of participants (i.e. $HbA1c_{SA}$-$HbA1c_{SB}$ = $HbA1c_{NA}$-$HbA1c_{NB}$ in Table 2).

Patient group	Drug A	Drug B	Difference
In strata (S)	$HbA1c_{SA}$	$HbA1c_{SB}$	$HbA1c_{SA}$ - $HbA1c_{SB}$
Not in strata (N)	$HbA1c_{NA}$	$HbA1c_{NB}$	$HbA1c_{NA}$ - $HbA1c_{NB}$

For peer review only

Table 3. Protocol Amendments

Protocol Amendments	
**SA1** v3 06.07.16	Amendment to randomisation process to allocate individual bottles rather than 'packs' of 3 bottles to allow for shorter expiry dates, and clarification of safety reporting procedures
**SA4** v4 20.03.17	Amendment to exclusion criteria to allow patients who have previously tried the study drugs to be included, as long as this has not been in the previous 3 months. The original criteria was unnecessarily strict and did not reflect real-world prescribing habits. The amendment also removed the blanket exclusion for patients in concurrent clinical trials, providing sufficient washout period between IMPs.
**SA6** v5 01.08.17	Amendment to eligibility criteria to include patients taking metformin-only, or metformin and a sulfonylurea. This was adjusted due to the change in guidelines and prescribing trends leading to decline in use of sulfonylureas. At the time of study design sulfonylureas were the most commonly prescribed second line therapy in the UK. Subsequent decline in their use in favour of DPP4-inhibitors and SGLT2 inhibitors (2), resulted in the inclusion of patients currently treated with either metformin and sulfonylureas or metformin only. We will perform a sensitivity analysis to determine if the difference in study "epoch" (before/after this amendment) has any impact on the main study outcomes.<br>Altered exclusion criteria also added 'limb ischaemia' due to updated safety information for Canagliflozin, and an upper limit of HbA1c >110mmol/mol.
**SA9** v6 15.05.18	Amendment to sample size due to over-cautious sample calculations (alpha changed to 0.05), extension to recruitment period due to delays in regulatory approvals at study set-up and slow early recruitment, and additional secondary analysis included on the advice of the Data Monitoring Committee.
**SA10** v7 22.02.19	Amendment to study analysis plan. Following advice from the Trial Steering Committee statistician, the protocol was amended to analyse only those completing at least 12 weeks on therapy, as this will determine whether the strata result in differences in response (we cannot adequately measure glycaemic response by HbA_{1c} if the patient has been on the drug for less than 12 weeks). A separate analysis will be performed to determine whether the strata influence tolerability by assessing whether the proportion completing at least 12 weeks on therapy differs by drug and strata.
**SA12** v8 20.03.20	Amendment to ensure ongoing participant safety and study integrity during Covid-19 pandemic. Urgent safety measures included (i) extension of visit windows to 14-18 weeks to allow greater flexibility for participants who are unwell/isolating, (ii) provision for remote visits with sample collection outside the usual research setting, (iii) ensuring participants remained on study therapy when only a remote visit is possible, by allowing an additional 'continuation' bottle of the same IMP to be issued, or when no other option, transfer to the next IMP without collection of blood samples.

For peer review only - http://bmjopen.bmj.com/site/about/guidelines.xhtml

REFERENCES

1. Qaseem A, Barry MJ, Humphrey LL, Forciea M, for the Clinical Guidelines Committee of the American College of P. Oral pharmacologic treatment of type 2 diabetes mellitus: A clinical practice guideline update from the american college of physicians. Annals of Internal Medicine. 2017;166(4):279-90.
2. Dennis JM, Henley WE, McGovern AP, Farmer AJ, Sattar N, Holman RR, et al. Time trends in prescribing of type 2 diabetes drugs, glycaemic response and risk factors: A retrospective analysis of primary care data, 2010-2017. Diabetes, obesity & metabolism. 2019;21(7):1576-84.
3. Pearson ER. Personalized medicine in diabetes: the role of 'omics' and biomarkers. Diabetic Medicine. 2016;33(6):712-7.
4. Davies MJ, D'Alessio DA, Fradkin J, Kernan WN, Mathieu C, Mingrone G, et al. Management of hyperglycaemia in type 2 diabetes, 2018. A consensus report by the American Diabetes Association (ADA) and the European Association for the Study of Diabetes (EASD). Diabetologia. 2018;61(12):2461-98.
5. Jones AG, McDonald TJ, Shields BM, Hill AV, Hyde CJ, Knight BA, et al. Markers of beta-Cell Failure Predict Poor Glycemic Response to GLP-1 Receptor Agonist Therapy in Type 2 Diabetes. Diabetes care. 2016;39(2):250-7.
6. DeFronzo RA, Stonehouse AH, Han J, Wintle ME. Relationship of baseline HbA1c and efficacy of current glucose-lowering therapies: a meta-analysis of randomized clinical trials. Diabetic Medicine. 2010;27(3):309-17.
7. Thong KY, McDonald TJ, Hattersley AT, Blann AD, Ramtoola S, Duncan C, et al. The association between postprandial urinary C-peptide creatinine ratio and the treatment response to liraglutide: a multi-centre observational study. Diabetic Medicine. 2014;31(4):403-11.
8. Bihan H, Ng WL, Magliano DJ, Shaw JE. Predictors of efficacy of GLP-1 agonists and DPP-4 inhibitors: A systematic review. Diabetes Research and Clinical Practice. 2016;121:27-34.
9. Dennis JM. Precision medicine in type 2 diabetes: Using individualised prediction models to optimise selection of treatment. Diabetes. 2020;(in press).
10. Dennis JM, Henley WE, Weedon MN, Lonergan M, Rodgers LR, Jones AG, et al. Sex and BMI Alter the Benefits and Risks of Sulfonylureas and Thiazolidinediones in Type 2 Diabetes: A Framework for Evaluating Stratification Using Routine Clinical and Individual Trial Data. Diabetes care. 2018;41(9):1844-53.
11. Dennis JM, Shields BM, Hill AV, Knight BA, McDonald TJ, Rodgers LR, et al. Precision Medicine in Type 2 Diabetes: Clinical Markers of Insulin Resistance Are Associated With Altered Short- and Long-term Glycemic Response to DPP-4 Inhibitor Therapy. Diabetes care. 2018;41(4):705-12.
12. Rodgers L, ER. P, Hammersley S, Angwin C, McDonald TJ, Shields BM, et al. Patients with a high fasting glucose respond better to sulphonylureas than dipeptidylpeptidase IV (DPP-IV) inhibitors: a MASTERMIND study. Diabetic Medicine. 2015;32(S1):1-3.
13. Viberti G, Kahn SE, Greene DA, Herman WH, Zinman B, Holman RR, et al. A Diabetes Outcome Progression Trial (ADOPT). An international multicenter study of the comparative efficacy of rosiglitazone, glyburide, and metformin in recently diagnosed type 2 diabetes. 2002;25(10):1737-43.
14. Drucker DJ, Nauck MA. The incretin system: glucagon-like peptide-1 receptor agonists and dipeptidyl peptidase-4 inhibitors in type 2 diabetes. The Lancet. 2006;368(9548):1696-705.
15. Tahrani AA, Barnett AH, Bailey CJ. SGLT inhibitors in management of diabetes. The Lancet Diabetes & Endocrinology. 2013;1(2):140-51.
16. Chan A-W, Tetzlaff JM, Altman DG, Laupacis A, Gøtzsche PC, Krleža-Jerić K, et al. SPIRIT 2013 statement: defining standard protocol items for clinical trials. Annals of internal medicine. 2013;158(3):200-7.

For peer review only - http://bmjopen.bmj.com/site/about/guidelines.xhtml

17. Merck Sharp D. Summary of Product Characteristics: Januvia (sitagliptin phosphate monohydrate) available at https://www.medicines.org.uk/emc/product/7887/smpc. Electronic Medicines Compendium (EMC) 2020.
18. Napp Pharmaceuticals L. Summary of Product Characterstics: Invokana (Canagliflozin Hemihydrate) available at https://www.medicines.org.uk/emc/product/8855/smpc. Electronic Medicines Compendium (EMC). 2020.
19. Takeda. Summary of Product Characteristics: Actos Tablets available at https://www.medicines.org.uk/emc/product/1287/smpc. Electronic Medicines Compendium (EMC) 2019.
20. WHO. Use of glycated haemoglobin (HbA1c) in the diagnosis of diabetes mellitus. 2011. www.who.int/diabetes/publications/report-hba1c_2011.pdf.
21. Lund SS, Tarnow L, Stehouwer CD, Schalkwijk CG, Frandsen M, Smidt UM, et al. Targeting hyperglycaemia with either metformin or repaglinide in non-obese patients with type 2 diabetes: results from a randomized crossover trial. Diabetes, obesity & metabolism. 2007;9(3):394-407.
22. Dwan K, Li T, Altman DG, Elbourne D. CONSORT 2010 statement: extension to randomised crossover trials. BMJ. 2019;366:l4378.

For peer review only

For peer review only - http://bmjopen.bmj.com/site/about/guidelines.xhtml

TriMaster CONSORT Flow Diagram

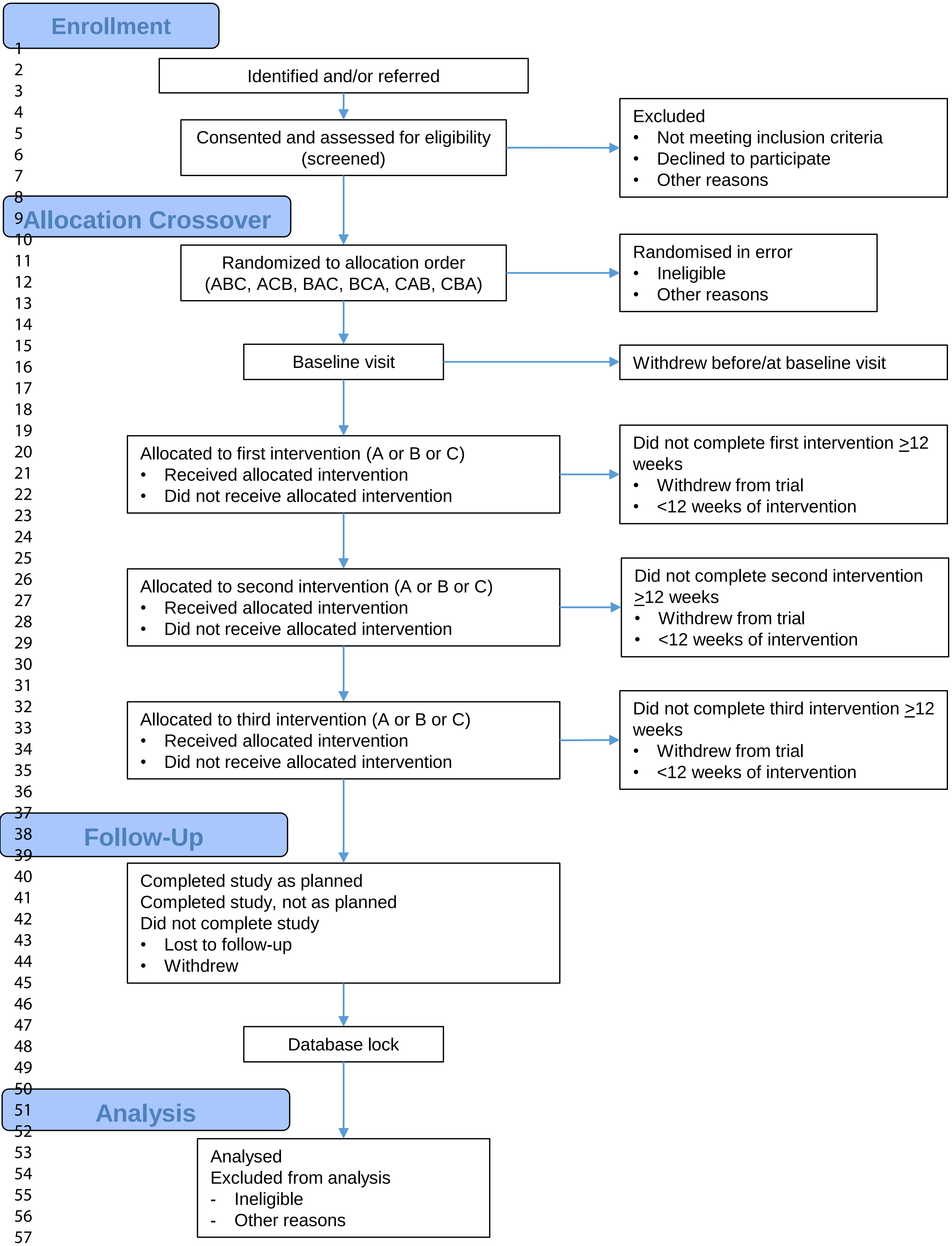

For peer review only - http://bmjopen.bmj.com/site/about/guidelines.xhtml

Figure 2. TriMaster Schedule of Assessment

	STUDY PERIOD							
	Pre-	Screening	Baseline	Treatment			Follow up	
**TIMEPOINT****	*-t*$_1$	*t*$_0$	t$_1$ v1	*t*$_2$ v2	*t*$_3$ v3	*t*$_4$ v4	*t*$_5$ v5	*t*$_6$
**ENROLMENT:**								
Informed consent		X						
Medical History		X						
Concomitant medication		X						
Foot pulses		X						
Weight		X						
Height		X						
Eligibility Bloods		X	X[1]					
Urine Dip		X	X[1]					
Pregnancy Test (WOCBP)		X						
Randomisation			X					
**INTERVENTIONS:**								
Medication dispensing			X	X	X	X		
Medication return				X	X	X		
IMP accountability				X	X	X		
First IMP A/B/C				↔				
Second IMP A/B/C					↔			
Third IMP A/B/C						↔		
IMP code break (GP/patient)								X
**ASSESSMENTS:**								
Bloods (see below)			X	X	X	X		
Urine Dip			X	X	X	X		
Genital swabs[2]			X	X	X	X		
Urine collection			X	X	X	X		
Pregnancy Test (WOCBP)			X	X	X	X		
DTSQ			X	X	X	X		
Patient experience			X	X	X	X	X	
Family history of diabetes			X					
Concomitant medication			X	X	X	X		
Weight			X	X	X	X		
Waist & hip measurement			X	X	X	X		
Body fat %			X	X	X	X		
Blood pressure			X	X	X	X		
Oedema of leg/ankle			X	X	X	X		
Adherence			X	X	X	X		
Side Effects			X	X	X	X		
Adverse events review			X	X	X	X		
**BLOODS:**								
HbA1c		X	X	X	X	X		
Full blood count		X		X	X	X		
Creatinine		X	X	X	X	X		
ALT		X						
Glycosylated albumin			X	X	X	X		
Glucose			X[3]	X	X	X		
C-peptide			X[3]	X	X	X		
Insulin			X[3]	X	X	X		
AST			X	X	X	X		
Albumin			X	X	X	X		
Bilirubin			X	X	X	X		
GGT			X	X	X	X		
Triglycerides			X	X	X	X		
Total, HDL and LDL cholesterol			X	X	X	X		
Autoantibodies–GAD, IA2, ZnT8			X					
Medication Levels				X	X	X		
B-type Natriuretic Peptide (BNP)			X	X	X	X		

[1] Where baseline visit takes place more than 2 weeks after screening visit, eligibility blood samples must be repeated.
[2] Optional procedure for participants at sites which have previously agreed to sample collection
[3] Analysis performed on both Visit 1 Baseline and Visit 1 MMTT samples. Other analyses at Visit 1 performed on Baseline only

For peer review only

Reporting checklist for protocol of a clinical trial – TriMaster

Based on the SPIRIT guidelines: Chan A-W, Tetzlaff JM, Altman DG, Laupacis A, Gøtzsche PC, Krleža-Jerić K, Hróbjartsson A, Mann H, Dickersin K, Berlin J, Doré C, Parulekar W, Summerskill W, Groves T, Schulz K, Sox H, Rockhold FW, Rennie D, Moher D. SPIRIT 2013 Statement: Defining standard protocol items for clinical trials. Ann Intern Med. 2013;158(3):200-207

		Reporting Item	Page Number
**Administrative information**			
Title	#1	Descriptive title identifying the study design, population, interventions, and, if applicable, trial acronym	1
Trial registration	#2a	Trial identifier and registry name. If not yet registered, name of intended registry	2
Trial registration: data set	#2b	All items from the World Health Organization Trial Registration Data Set	Appendix 3
Protocol version	#3	Date and version identifier	16
Funding	#4	Sources and types of financial, material, and other support	15
Roles and responsibilities: contributorship	#5a	Names, affiliations, and roles of protocol contributors	1 & 14
Roles and responsibilities: sponsor contact information	#5b	Name and contact information for the trial sponsor	Appendix 3
Roles and responsibilities: sponsor and funder	#5c	Role of study sponsor and funders, if any, in study design; collection, management, analysis, and interpretation of data; writing of the report; and the decision to submit the report for publication, including whether they will have ultimate authority over any of these activities	15

For peer review only

Roles and responsibilities: committees	#5d	Composition, roles, and responsibilities of the coordinating centre, steering committee, endpoint adjudication committee, data management team, and other individuals or groups overseeing the trial, if applicable (see Item 21a for data monitoring committee)	14-15
**Introduction**			
Background and rationale	#6a	Description of research question and justification for undertaking the trial, including summary of relevant studies (published and unpublished) examining benefits and harms for each intervention	2
Background and rationale: choice of comparators	#6b	Explanation for choice of comparators	3
Objectives	#7	Specific objectives or hypotheses	4
Trial design	#8	Description of trial design including type of trial (eg, parallel group, crossover, factorial, single group), allocation ratio, and framework (eg, superiority, equivalence, non-inferiority, exploratory)	5
**Methods: Participants, interventions, and outcomes**			
Study setting	#9	Description of study settings (eg, community clinic, academic hospital) and list of countries where data will be collected. Reference to where list of study sites can be obtained	5
Eligibility criteria	#10	Inclusion and exclusion criteria for participants. If applicable, eligibility criteria for study centres and individuals who will perform the interventions (eg, surgeons, psychotherapists)	6
Interventions: description	#11a	Interventions for each group with sufficient detail to allow replication, including how and when they will be administered	9
Interventions: modifications	#11b	Criteria for discontinuing or modifying allocated interventions for a given trial participant (eg, drug dose	9

For peer review only

		change in response to harms, participant request, or improving / worsening disease)	
Interventions: adherance	#11c	Strategies to improve adherence to intervention protocols, and any procedures for monitoring adherence (eg, drug tablet return; laboratory tests)	10
Interventions: concomitant care	#11d	Relevant concomitant care and interventions that are permitted or prohibited during the trial	7
Outcomes	#12	Primary, secondary, and other outcomes, including the specific measurement variable (eg, systolic blood pressure), analysis metric (eg, change from baseline, final value, time to event), method of aggregation (eg, median, proportion), and time point for each outcome. Explanation of the clinical relevance of chosen efficacy and harm outcomes is strongly recommended	8
Participant timeline	#13	Time schedule of enrolment, interventions (including any run-ins and washouts), assessments, and visits for participants. A schematic diagram is highly recommended (see Figure)	Figures 1 & 2
Sample size	#14	Estimated number of participants needed to achieve study objectives and how it was determined, including clinical and statistical assumptions supporting any sample size calculations	8
Recruitment	#15	Strategies for achieving adequate participant enrolment to reach target sample size	11

Methods: Assignment of interventions (for controlled trials)

Allocation: sequence generation	#16a	Method of generating the allocation sequence (eg, computer-generated random numbers), and list of any factors for stratification. To reduce predictability of a random sequence, details of any planned restriction (eg, blocking) should be provided in a separate document that is unavailable to those who enrol participants or assign interventions	9

For peer review only

Allocation concealment mechanism	#16b	Mechanism of implementing the allocation sequence (eg, central telephone; sequentially numbered, opaque, sealed envelopes), describing any steps to conceal the sequence until interventions are assigned	10
Allocation: implementation	#16c	Who will generate the allocation sequence, who will enrol participants, and who will assign participants to interventions	10
Blinding (masking)	#17a	Who will be blinded after assignment to interventions (eg, trial participants, care providers, outcome assessors, data analysts), and how	10
Blinding (masking): emergency unblinding	#17b	If blinded, circumstances under which unblinding is permissible, and procedure for revealing a participant's allocated intervention during the trial	10

Methods: Data collection, management, and analysis

Data collection plan	#18a	Plans for assessment and collection of outcome, baseline, and other trial data, including any related processes to promote data quality (eg, duplicate measurements, training of assessors) and a description of study instruments (eg, questionnaires, laboratory tests) along with their reliability and validity, if known. Reference to where data collection forms can be found, if not in the protocol	Figure 2 & pages 7 & 11
Data collection plan: retention	#18b	Plans to promote participant retention and complete follow-up, including list of any outcome data to be collected for participants who discontinue or deviate from intervention protocols	10
Data management	#19	Plans for data entry, coding, security, and storage, including any related processes to promote data quality (eg, double data entry; range checks for data values). Reference to where details of data management procedures can be found, if not in the protocol	12
Statistics: outcomes	#20a	Statistical methods for analysing primary and secondary outcomes. Reference to where other details of the	13

For peer review only

		statistical analysis plan can be found, if not in the protocol	
Statistics: additional analyses	#20b	Methods for any additional analyses (eg, subgroup and adjusted analyses)	14
Statistics: analysis population and missing data	#20c	Definition of analysis population relating to protocol non-adherence (eg, as randomised analysis), and any statistical methods to handle missing data (eg, multiple imputation)	14

Methods: Monitoring

Data monitoring: formal committee	#21a	Composition of data monitoring committee (DMC); summary of its role and reporting structure; statement of whether it is independent from the sponsor and competing interests; and reference to where further details about its charter can be found, if not in the protocol. Alternatively, an explanation of why a DMC is not needed	14
Data monitoring: interim analysis	#21b	Description of any interim analyses and stopping guidelines, including who will have access to these interim results and make the final decision to terminate the trial	NA
Harms	#22	Plans for collecting, assessing, reporting, and managing solicited and spontaneously reported adverse events and other unintended effects of trial interventions or trial conduct	14
Auditing	#23	Frequency and procedures for auditing trial conduct, if any, and whether the process will be independent from investigators and the sponsor	14

Ethics and dissemination

Research ethics approval	#24	Plans for seeking research ethics committee / institutional review board (REC / IRB) approval	2

For peer review only

Protocol amendments	#25	Plans for communicating important protocol modifications (eg, changes to eligibility criteria, outcomes, analyses) to relevant parties (eg, investigators, REC / IRBs, trial participants, trial registries, journals, regulators)	16
Consent or assent	#26a	Who will obtain informed consent or assent from potential trial participants or authorised surrogates, and how (see Item 32)	11
Consent or assent: ancillary studies	#26b	Additional consent provisions for collection and use of participant data and biological specimens in ancillary studies, if applicable	NA
Confidentiality	#27	How personal information about potential and enrolled participants will be collected, shared, and maintained in order to protect confidentiality before, during, and after the trial	12
Declaration of interests	#28	Financial and other competing interests for principal investigators for the overall trial and each study site	16
Data access	#29	Statement of who will have access to the final trial dataset, and disclosure of contractual agreements that limit such access for investigators	14
Ancillary and post trial care	#30	Provisions, if any, for ancillary and post-trial care, and for compensation to those who suffer harm from trial participation	NA
Dissemination policy: trial results	#31a	Plans for investigators and sponsor to communicate trial results to participants, healthcare professionals, the public, and other relevant groups (eg, via publication, reporting in results databases, or other data sharing arrangements), including any publication restrictions	15
Dissemination policy: authorship	#31b	Authorship eligibility guidelines and any intended use of professional writers	NA
Dissemination policy: reproducible research	#31c	Plans, if any, for granting public access to the full protocol, participant-level dataset, and statistical code	15

Appendices

For peer review only

Informed consent materials	#32	Model consent form and other related documentation given to participants and authorised surrogates	Appendix 2
Biological specimens	#33	Plans for collection, laboratory evaluation, and storage of biological specimens for genetic or molecular analysis in the current trial and for future use in ancillary studies, if applicable	Figure 2 & page 7

The SPIRIT checklist is distributed under the terms of the Creative Commons Attribution License CC-BY-ND 3.0. This checklist can be completed online using https://www.goodreports.org/, a tool made by the EQUATOR Network in collaboration with Penelope.ai

For peer review only

Sponsored by the Royal Devon and Exeter NHS Foundation Trust

**Site Logo(s)**

STUDY NAME: **TRIMASTER**

Screening ID:	Study ID:	DOB
		/ / 19

**CONSENT STATEMENTS**	**Please circle**	**Initials**
I have been given study information leaflet [Version [ ] dated [ ]] and supplementary information about the treatments used in this study. I have had the opportunity to ask questions and have had these answered satisfactorily.	YES/NO	
I am happy to:<br>• attend 6 appointments, having fasted overnight for 4 of these.<br>• try 3 different regularly prescribed diabetes treatments for up to 16 weeks each.<br>• provide information about my diabetes for use in this project.<br>• allow the research team to contact my clinicians/GP about my diabetes treatment and study participation, and to provide them with clinical results relevant to my care.<br>• provide blood and urine samples for use by this study.	YES/NO	
I understand that:<br>• my participation is voluntary and that I may withdraw at any time without my clinical care being affected.<br>• individuals from the study team, regulatory authorities or the NHS Trust will have access to relevant sections of my medical notes and data collected during the study for research, monitoring and audit purposes.	YES/NO	
**OPTIONAL CONSENT STATEMENTS**		
I agree that DNA may be extracted from samples for the purpose of this project	YES/NO	
I am happy to provide self-collected genital swabs (Exeter site only).	YES/NO	
I am happy to gift samples and data from the project to the Peninsula Research Bank in Exeter to be used for future research.	YES/NO	
I agree that information held by the NHS and in my medical records may be used to follow up on my future health status.	YES/NO	
I am happy to be contacted by my local research team about participating in other future studies.	YES/NO	

**Participant Name**		**Signed**		**Date**
				/ /
I confirm that I am on the delegation log for the TriMaster study to obtain consent. In my opinion the participant understands what this study involves and has capacity to take part.				
**Name of Person Obtaining Consent**		**Signed**		**Date**
				/ /
**Clinician Confirming Eligibility***		**Signed**		**Date**
				/ /

* Eligibility (eligible/non-eligible) confirmed as indicated on the screening CRF
When completed: 1 for participant; 1 for researcher site file; 1 to be kept in medical notes (front page only)

For peer review only
For peer review only - http://bmjopen.bmj.com/site/about/guidelines.xhtml

Please stick consent barcode label below:

Visit 1 consent barcode	Visit 2 consent barcode
Visit 3 consent barcode	Visit 4 consent barcode

For peer review only

Data Category	Information
Primary registry and trial identifying number	ClinicalTrials.gov NCT02653209
Date of registration in primary registry	8 January 2016
Secondary identifying numbers	EudraCT 2015-002790-38; ISRCTN12039221; Sponsor 1603221; Funder MR/N00633X/1; HRA 16/SC/0147; IRAS 183044
Source(s) of monetary or material support	Medical Research Council, UK
Primary sponsor	Royal Devon and Exeter NHS Foundation Trust
Sponsor contact details	Alison Kerridge, Assistant R&D Manager;<br>Research and Development,<br>First Floor, Bowmoor House<br>Barrack Road,<br>Exeter<br>EX2 5DW<br>alison.kerridge@nhs.net; 01392 403055
Contact for public queries	Catherine Angwin, Trial Manager;<br>NIHR Exeter Clinical Research Facility<br>Room 02.15, RILD Building<br>Barrack Road<br>Exeter<br>EX2 5DW<br>Rde-tr.DiabetesDrugResponse@nhs.net; 01392 408180
Contact for scientific queries	Professor Andrew Hattersley FRCP FMedSci FRS, Chief Investigator;<br>University of Exeter Medical School<br>RILD Building<br>Barrack Road<br>Exeter<br>EX2 5DW<br>A.T.Hattersley@exeter.ac.uk; 01392 408260
Public title	TriMaster: Study of a DPP4 inhibitor, SGLT2 Inhibitor and Thiazolidinedione as Third Line Therapy in patients with Type 2 Diabetes.
Scientific title	TriMaster: Randomised Double-Blind Crossover Study of a DPP4 Inhibitor, SGLT2 Inhibitor and Thiazolidinedione as Third Line Therapy in Patients With Type 2 Diabetes Who Have Suboptimal Glycaemic Control on Dual Therapy with Metformin and a Sulphonylurea
Countries of recruitment	United Kingdom
Health condition(s) or problem(s) studied	Type 2 Diabetes
Intervention(s)	Drug: Sitagliptin (2x50mg as over-encapsulated hard shell to be taken orally, once a day); Drug: Canagliflozin (100mg as over-encapsulated hard shell to be taken orally, once a day); Drug: Pioglitazone (30mg as over-encapsulated hard shell to be taken orally, once a day)
	All participants receive all 3 interventional treatments for 16 weeks each in random order according to one of 6 possible treatment order (ABC, ACA, BAC, BCA, CAB, CBA) with no washout period
Key inclusion and exclusion criteria	Ages eligible for study: 30 – 80 years (inclusive); Sexes eligible for study: both; Accepts healthy volunteers: no

For peer review only

	Inclusion Criteria: Clinical diagnosis of Type 2 diabetes, Age ≥30 and ≤80, currently treated with two classes of oral glucose-lowering therapy that do not include a DPP4-inhibitor, a SGLT2 inhibitor or a thiazolidinedione, diabetes duration ≥12 months, no change in diabetes treatment (new treatments or dose change) within previous 3 months, HbA1c >58mmol/mol (7.5%) and ≤110mmol/mol (12.2%) – confirmed at screening visit, eGFR ≥60mls/min/1.73m^{2} – confirmed at screening visit, able and willing to give informed consent
	Exclusion Criteria: Changes in glucose-lowering therapy or dose within last 3 months, HbA1c ≤58mmol/mol (7.5%) or >110mmol/mol (12.2%), eGFR <60mls/min/1.73m^{2}, diabetes duration <12 months, ALT >2.5 x upper limit of the assay normal range or known liver disease, specifically >30µmol/L that is associated with other evidence of liver failure, insulin treated within the last 12 months, limb ischaemia shown by absence of both pulses in one or both feet, currently treated with corticosteroids, currently treated with rifampicin, gemfibrozil, phenytoin and carbamazepine, active infection (requiring antibiotics at present), foot ulcer requiring antibiotics within previous three months, recent (within 3 months) significant surgery or planned surgery (excluding minor procedures), acute cardiovascular episode (angina, myocardial infarction, stroke, transient ischemic episode) occurring within the previous 3 months, history of heart failure, current use of loop diuretic therapy (Furosemide or Bumetanide), history of bladder carcinoma, current/ongoing investigation for macroscopic haematuria, history of diabetic ketoacidosis, history of pancreatitis, pregnant, breastfeeding or planning a pregnancy over the study period, concurrent participation on another CTIMP (where IMP is currently being taken or without sufficient washout period), unable or unwilling to give informed consent
Study type	Interventional
	Allocation: Randomised; Intervention model: Crossover assignment; Masking: Double (Participant, Investigator)
	Primary Purpose: Treatment
	Phase 4
Date of first enrolment	1 November 2016
Target sample size	525 (reduced from 600)
Recruitment status	Active, not recruiting
Primary outcome(s)	On treatment HbA1c in obese patients (BMI>30kgm^{-2}) compared to non-obese patients (BMI<30kgm^{-2}), (time frame: 16 weeks); On treatment HbA1c in patients with

For peer review only

	an eGFR<90mls/min/1.73m^2 compared to patients with an eGFR>90mls/min/1.73m^2, (time frame: 16 weeks)
Key secondary outcomes	Patient preference [Time frame: 48-54 weeks (3x16 weeks of therapy)]; Prevalence of side effects [Time frame: 48-54 weeks (3x16 weeks of therapy)]; HbA1c on therapy against predefined test of gender heterogeneity (Time frame: 16 weeks)
Ethics Review	Status: Approved
	Date of Approval: 9 May 2016
	South Central – Oxford A Research Ethics Committee Nrescommittee.southcentral-oxforda@nhs.net
Completion Date	January 2021
Summary Results	N/A as study has not completed yet

For peer review only
