# Supplementary material for: TriMaster: randomised double-blind crossover study of a DPP4 inhibitor, SGLT2 inhibitor and thiazolidinedione as second-line or third-line therapy in patients with type 2 diabetes who have suboptimal glycaemic control on metformin treatment with or without a sulfonylurea—a MASTERMIND study protocol
Source: BMJ Open. 2020 Dec 21;10(12):e042784. doi: 10.1136/bmjopen-2020-042784 (PMC7754630; doi:10.1136/bmjopen-2020-042784)
Supplement: Reviewer comments [file bmjopen-2020-042784.reviewer_comments.pdf]

PEER REVIEW HISTORY

BMJ Open publishes all reviews undertaken for accepted manuscripts. Reviewers are asked to complete a checklist review form (**http://bmjopen.bmj.com/site/about/resources/checklist.pdf**) and are provided with free text boxes to elaborate on their assessment. These free text comments are reproduced below.

ARTICLE DETAILS

**TITLE (PROVISIONAL)**	TriMaster: Randomised Double-Blind Crossover study of a DPP4 inhibitor, SGLT2 inhibitor and thiazolidinedione as second or third line therapy in patients with Type 2 diabetes who have suboptimal glycaemic control on metformin treatment with or without a sulfonylurea – a MASTERMIND study protocol
**AUTHORS**	Angwin, Catherine; Jenkinson, C; Jones, Angus; Jennison, Christopher; Henley, W; Farmer, Andrew; Sattar, Naveed; Holman, Rury; Pearson, Ewan; Shields, Beverley; Hattersley, Andrew

VERSION 1 – REVIEW

**REVIEWER**	MJB van Baar<br>Amsterdam University Medical Centers - location VUmc
**REVIEW RETURNED**	21-Aug-2020

**GENERAL COMMENTS**	No further comments
